# Effects of second responder programs on repeat incidents of family abuse: An updated systematic review and meta‐analysis

**DOI:** 10.1002/cl2.1217

**Published:** 2022-01-28

**Authors:** Kevin Petersen, Robert C. Davis, David Weisburd, Bruce Taylor

**Affiliations:** ^1^ Department of Criminology, Law and Society George Mason University Fairfax Virginia USA; ^2^ National Police Foundation Arlington Virginia USA; ^3^ Institute of Criminology, Faculty of Law Hebrew University Jerusalem Israel; ^4^ NORC at the University of Chicago Bethesda Maryland USA

## Abstract

**Background:**

Family abuse is a recurrent phenomenon within a select population of households. This form of abuse can include any physical or psychological harassment that occurs between family or household members, and often involves complex mental and emotional issues that are resistant to intervention. Traditional criminal justice strategies for combating this issue have evolved over time but have frequently demonstrated limited success. Within the past few decades, multiagency programs to address repeat family abuse have gained popularity. One such program, termed “second responders,” teams police officers with social service workers, victim advocates, or counselors to conduct follow‐up visits with victims of family abuse following a complaint. Second responders seek to educate victims about the cyclical nature of family abuse, engage in safety planning, and/or provide service referrals. These interventions are based on the premise that victims are more likely to be receptive to crime prevention opportunities immediately following victimization. Second responder interventions have received support from the US Department of Justice and their adoption has spread in both the United States and internationally, however, there remains little conclusive evidence on their effects.

**Objectives:**

To update and extend the findings of the prior second responders systematic review and meta‐analysis by synthesizing the results of published and unpublished second responder evaluations through October of 2021. This review also examines the use of victim services as a secondary outcome and incorporates a number of additional moderator analyses.

**Search Methods:**

The Global Policing Database (GPD), a repository of all experimental and quasi‐experimental evaluations of policing interventions conducted since 1950, was searched using keywords related to second responder interventions and repeat family violence from 2004 to December 2019 (https://gpd.uq.edu.au/s/gpd/page/about). This search was also supplemented with additional strategies, such as reference harvesting of prior reviews, searching 2020 and 2021 volumes of leading academic journals, reviewing the reference lists of eligible studies, searching additional gray literature repositories focused on domestic violence, and consulting with eligible study authors.

**Selection Criteria:**

Eligible studies were required to include a treatment group that received the second responder intervention and a comparison group that did not. Assignment to these conditions could be either experimental or quasi‐experimental, but quasi‐experimental studies were required to use either matched comparison groups or multivariate analysis methods to control for confounding factors. Eligibility was limited to studies reporting on at least one measure of repeat family abuse, such as intimate partner violence, elder abuse, or general family abuse. Measures of repeat abuse could be based on either official (i.e., police data) or unofficial (i.e., victim survey data) data sources.

**Data Collection and Analysis:**

Five new studies were identified between 2004 and 2019, all of which contained sufficient data for the calculation of at least one effect size. Along with the 10 studies included in the prior review, a total of 15 studies and 29 distinct effect sizes were analyzed across three outcome constructs. Effect sizes were calculated as logged odds ratios and results were synthesized using random effects models with restricted maximum likelihood estimation. Final results were exponentiated to represent the percentage point difference in the odds of a given outcome for treatment groups relative to control groups. Risk of bias was assessed using items adapted from the Cochrane Risk of Bias tools for experimental and quasi‐experimental studies. Eligible studies were generally considered to be of low risk of bias, however, issues with survey success/contact rates and the analytical approaches to these problems led to concern in several studies.

**Results:**

These analyses suggest that second responder interventions produced no significant effects on either police or victim‐reported measures of repeat family abuse, in aggregate. However, findings from the more rigorous experimental studies indicated that second responder interventions were associated with a statistically significant 22% (95% confidence interval [CI] [1.04, 1.43]) increase in the odds of a police‐reported repeat family abuse incident, with no significant variability in individual study results. Additionally, studies that measured the use of victim services as a secondary outcome were associated with a statistically significant 9% (95% CI [1.02, 1.16]) increase in the odds of service use for treatment groups relative to control groups. Several study characteristics also proved to be important moderators of treatment effects. Increases in the speed of the second response were associated with significant decreases in the odds of a victim‐reported repeat incident, and studies that measured repeat family abuse using households were associated with significantly higher odds of a police‐reported repeat incident, compared to studies that used the same victim or victim/offender pairing more generally.

**Authors' Conclusions:**

Second responder interventions are undoubtedly appealing based on their logic and intentions. Yet, well‐intentioned programs with sound logic can still backfire, and the results of this updated review provide evidence that may be suggestive of a backfire effect. Even so, any firm conclusions from this review are limited by a lack of knowledge on the mechanisms operating in between the implementation of the second response intervention and the observed effects, as well as the small sample sizes involved in many analyses. While it seems clear that these programs are not producing any broad reductions in self‐reported victimization, the increase in police‐reported violence seen in experimental studies could indicate either a true increase in abuse or an increased willingness to call the police. The lack of observed impact on victim‐reported violence would suggest the latter, but without more specific measures, such conclusions should be avoided. If these results are indicative of increased reporting, however, many may consider this a desirable outcome, particularly given the often‐underreported nature of family abuse and the potential for increased reporting to lead to long‐term reductions in abuse. Furthermore, these results provide an indication that second responder programs can produce other intended effects, such as increasing the retention of victim services, and that the specific characteristics of these interventions may moderate their effects. It is unclear why elements such as the immediacy of the second response or the unit of analysis being evaluated would impact study results, but these observations are consistent with the theory that domestic violence interventions must capitalize on short windows of opportunity and create separation between victims and offenders to reduce exposure and subsequent victimization. This potential indicates a need for more research on second responder programs, but specifically research that examines these moderating characteristics and mechanisms. Even in light of this potential, second responder programs do not, on average, appear to reduce the prevalence of repeat family abuse. Given the presence of alternative (and possibly more effective) domestic violence interventions that now exist (e.g., Safe Dates, Shifting Boundaries, Green Dot, etc.), it seems that policymakers may wish to look elsewhere for efforts to reduce family abuse.

## 1 PLAIN LANGUAGE SUMMARY

### 1.1 Second responder interventions are not associated with reductions in repeat family abuse, though certain characteristics deserve further research

Second responder interventions are multi‐agency approaches to family abuse reduction that team police officers with social service workers, victim advocates, or domestic violence counselors. Second responder programs do not produce significant reductions in victim‐reported repeat family abuse. Rigorous experimental studies suggest that these programs may in fact lead to significant increases in police‐reported repeat abuse.

More immediate responses are associated with greater reductions in victim‐reported repeat abuse. Studies analyzing households rather than victims or victim/offender pairings report higher odds of police‐reported repeat abuse. Additionally, evidence suggests that second responder interventions increase the usage of domestic violence services.

### 1.2 What is this review about?

Second responder programs are intended to provide victims with the knowledge and resources needed to reduce their chances of future victimization. Second responder teams attempt to contact victims shortly after a domestic complaint to provide education, safety planning, and services referrals. They also warn abusers about the potential for punishment.

### 1.3 What is the aim of this review?

This updated Campbell systematic review and meta‐analysis examines the efficacy of second responder interventions for reducing repeat family abuse and increasing the use of victim services. It summarizes and synthesizes the results of 15 studies, mostly from the USA.

### 1.4 What studies are included?

Eligibility for this review was limited to experimental and non‐equivalent control group quasi‐experimental designs. Eligible studies had to include a treatment group that received the second response intervention and to report on at least one outcome measure of repeat family abuse, such as intimate partner violence, elder abuse, or general family abuse.

Our search yielded 15 studies corresponding to 29 independent effect sizes across three outcome constructs. Fourteen studies were conducted in the USA and one study in the UK.

### 1.5 What are the main findings of the review

In aggregate, second responder programs do not appear to impact either police or victim‐reported repeat family abuse.

More rigorous experimental studies, however, indicate that these programs significantly increase the likelihood of a police‐reported repeat incident.

Evidence from a small number of studies suggests that victims may be significantly more likely to access services following contact with second responders.

Responses that occurred shortly after an incident led to decreases in victim‐reported incidents, while delayed responses led to increases in victim‐reported incidents. This finding is preliminary, however, and more research is needed.

Finally, evaluations of second responder programs appear more likely to report increased police‐reported abuse when following the same household over time, as opposed to the same victim or victim/offender pairing more generally.

### 1.6 What do the findings of the review mean?

The findings of this review question the efficacy of second responder programs. However, the desirability of these findings, and the factors that may be responsible for them, are also subject to debate. Evidence of increased reporting to the police but not increased reporting on victim surveys may indicate improvement in victims’ confidence and willingness to contact police. While this is not the long‐term goal of second responder programs, family abuse is frequently underreported and thus some may consider these findings to be favorable. It is also possible that an increased willingness to contact police in the short term may lead to long‐term reductions in abuse, though this claim is speculative.

Additionally, the findings of this review point to specific characteristics of second responder programs that may moderate their effectiveness. Second responders may need to contact victims as quickly as possible after a domestic incident to reduce repeat victimization, or encourage long‐term separation between the victim and abuser to reduce exposure.

Research is needed with specific focus on these underlying mechanisms. However, considering the tenuous nature of even our most optimistic findings, policymakers may need to seriously consider whether second responder programs are worthy of investment.

### 1.7 How up‐to‐date is this review?

This updated review employed search strategies intended to capture eligible studies through October 2021.

## 2 BACKGROUND

### 2.1 Policing repeat family abuse

Family abuse, or any form of physical or psychological abuse or aggression that occurs between family or household members, is a major criminal justice and public health concern. It has been estimated that one out of every three women will be abused by a family or household member during their life course (Garcia‐Moreno et al., 2013; Mizrachi, 2019), and that many will be victimized by an intimate partner (Smith et al., 2017). The consequences of family violence are also far‐reaching. Victims are often subjected to severe trauma which may lead to mental and physical health issues, depression, and suicidal ideations (Alejo, 2014). These effects can carry over to other family members as well, leading to issues with early childhood behavior and adjustment (English et al., 2003; Levendosky & Graham‐Bermann, 2001). Yet, official measures of family abuse are also severe underestimates of the true prevalence rate, and as many as half of all domestic incidents potentially go unreported to the police (Felson & Paré, 2005; Reaves, 2017).

The literature on desistance from family abuse suggests that the typical batterer's career is either short or sporadic: It has consistently been found that two in three households that report a domestic incident to the police do not report a subsequent incident over the following 6–12 months (see, e.g., Dowling & Morgan, 2019; Maxwell et al., 2010). For households that do report subsequent incidents, however, these incidents tend to be recurrent (Pate et al., 1992; Sherman, 2018). Early estimates indicated that an average of approximately eight repeat domestic abuse incidents may occur within victimized households over a 12‐month period (Straus et al., 1980). These households also tend to account for a disproportionate number of domestic homicides. In a 1977 study (see Wilt et al., 1977), nearly one‐third of domestic homicides could be attributed to households that had reported a previous domestic disturbance. More recently, research has suggested that at least one in four victims who experience a domestic abuse incident will be revictimized within 6 months (Boxall & Morgan, 2020; see also Goodin & Dunn, 2010), and that police are called to the households of over 50% of intimate partner homicide victims in the year before their death (see Campbell et al., 2003).

Criminal justice responses to repeat family abuse have evolved over time, from a general reluctance to consider family abuse as a pressing criminal justice issue, to mandating arrest for the predominate physical aggressor, to more holistic and coordinated multi‐agency approaches (see Mazerolle et al., 2018; Pate et al., 1992; Sherman, 2018). For those batterers who chronically abuse family members, it is no longer assumed that the initial police patrol response is sufficient in and of itself to protect victims from repeat abuse. Experts have come to realize that legal sanctions or victim actions that raise the personal or social costs to the batterer may promote a reduction or cessation in abuse (Fagan, 1989; Felson et al., 2005), but that legal sanctions alone are often unsuccessful (see Hilton et al., 2008). Effective solutions to family abuse (including intimate partner abuse, abuse within families or households, and elder abuse) must involve efforts to educate victims about their options and connect them with counseling, relocation, civil legal assistance, mental health, and other services that can lessen dependence on the abuser. Additionally, for these solutions to be effective they may need to occur shortly after a violent incident, when the risk of repeat victimization is at its highest (Sherman, 1997).

### 2.2 Second responder programs

Beginning in the 1970s in Ontario, Canada, a multidisciplinary intervention for repeat family abuse was developed with the goal of immediate intervention at incidents of family abuse, as well as to provide victims with support and services beyond that of the traditional criminal justice response (Jaffe et al., 1984). As Greenspan et al. (2005) note, early evaluations of this program reported optimistic findings, leading to the establishment of similar programs in the United Kingdom and the United States during the 1980s and 1990s. In the United Kingdom, this program was termed Domestic Violence Matters (DVM) and involved the provision of crisis intervention services to domestic violence victims in the immediate aftermath of an incident (Greenspan et al., 2005; Kelly et al., 1999). In the United States, these programs largely originated with the Domestic Violence Intervention and Education Prevention Program (DVEIP) in New York City. The DVEIP involved teams of specially trained police officers and social service workers who conducted follow‐up home visits with victims of domestic abuse after receiving an incident report (Davis & Taylor, 1997).

Interventions such as the DVM and DVEIP have since become known as “second responder” programs, or programs in which specially trained domestic violence advocates visit homes where family abuse incidents were recently reported in attempt to establish long‐term solutions (e.g., see Dean et al., 2000; Mickish, 2002). Second responder programs are based on the premise that family abuse is recurrent and that victims are most likely to access services, leave their abusers, or take other measures to address their safety in the immediate aftermath of a violent incident. In other words, there is a “window of opportunity” during the first few hours or days after a crime during which victims feel vulnerable and are willing to seriously consider behavioral and lifestyle changes (Anderson et al., 1995; Davis & Smith, 1994; Messing et al., 2015; see also Scott et al., 2015).

In second response programs, teams usually consist of both police officers and social service workers, though these responses can also be conducted by specially trained police officers, victim advocates, counselors, or social service workers alone. In either case, the second responder(s) provides the victim with support in accessing services such as housing, legal assistance, mental health, and safety planning. The team may provide information on these services and inform victims of their legal options, or may even assist the victim by providing referrals, facilitating an initial contact with a service provider, helping the victim to complete applications, obtain a restraining order, or advocating on the victim's behalf. In some models, the team may also attempt to make contact with the offender if present during the visit. These conversations are generally aimed at educating the offender on the legal consequences of continued abuse.

### 2.3 How the intervention might work

Research has indicated that victims of family abuse are often unlikely to access support services or engage in other help‐seeking behaviors (Macy et al., 2005). These efforts may be inhibited by a general lack of awareness regarding service availability or the means to access these services (Patzel, 2006). Thus, the theory underlying second responder interventions is that, by helping victims to understand the cyclical nature of family abuse, develop a safety plan, obtain a restraining order, increase their knowledge about legal rights and options, and/or provide shelter placement or other relocation assistance, the victim may be more likely to leave their abuser or take the steps necessary to increase their personal safety. In other words, second responder programs seek to “empower victims to access social service, mental health, and advocacy measures, so that they can take action to reduce their risk for future victimization” (Scott et al., 2015, p. 276). A secondary aim of the intervention is also to establish greater independence through counseling, job training, public assistance, or other social service referrals. Finally, conversations with abusers are intended to ensure an understanding of the legal ramifications associated with assaulting an intimate partner, and to reiterate that further abuse will result in additional sanctions.

Koppensteiner et al. (2019) argue that second response teams may lower the cost of accessing support services for victims of family abuse. That is, second responder programs remove barriers that would otherwise impede victims from accessing these services. These barriers could include lack of knowledge about service program eligibility, application procedures, hours of operation, etc. They may also assist in the translation of these services and options to victims who speak a different language or suffer from low self‐efficacy, depression, or substance abuse issues (see Stover et al., 2009), factors that often prevent successful service retention (see Fugate et al., 2005). Thus, second responder programs may impact repeat family abuse through multiple mechanisms. The second responders (and/or the service programs that they encourage victims to access) could persuade victims to end their abusive relationships and/or separate from the abuser for at least some period of time. Alternatively, the services and educational resources provided to victims may enable them to better navigate and mitigate the risks of victimization while remaining in their relationships. These programs may also exert a deterrent effect on abusers directly, for example by better communicating the certainty and severity of punishment associated with repeat abuse, or indirectly by encouraging and supporting victims during the prosecutorial process.

### 2.4 Why it is important to do this review

The US Department of Justice has extensively funded second responder programs. In England and Wales, funding of follow‐up visits with victims is largely provided by local Police and Crime Commissioners. Despite rapidly gaining popularity in the United States and other countries as well (e.g., Scott et al., 2015; Smee, 2021), evidence regarding the effectiveness of second responder programs is mixed. While several studies have indicated that second responders can prevent repeat victimization (Casey et al., 2007; Hovell et al., 2006; Pate et al., 1992), others have cast doubt on these claims, even suggesting that these programs may increase the odds of a repeat incident (Davis & Medina, 2001; Hovell et al., 2006).

For example, a series of field tests carried out in New York City (Davis & Medina, 2001; Davis & Taylor, 1997; Taylor, n.d.) suggested possible iatrogenic effects of second response programs. A pooled analysis conducted by Davis et al. (2006) reanalyzed data from three separate field experiments, each testing the same intervention on somewhat different populations. The pooled analyses indicated that the interventions were associated with an increase in reporting of new abusive incidents not only to authorities (which could indicate simply greater confidence in the police), but also to research interviewers. While it is not clear what mechanisms might lead to these unintended effects, some have expressed concern that unannounced home visits may further aggravate domestic abusers, thereby increasing the propensity for revictimization (Casey et al., 2007; Davis & Taylor, 1997).

In this regard, it is important to consider that even the most well‐intentioned criminal justice interventions can generate backfire effects (McCord, 2003), making it increasingly important to examine these programs empirically before any widespread adoption or implementation. However, the original second responders review yielded inconclusive findings (see Davis et al., 2008), with results suggestive of significant increases in police‐reported repeat family abuse, but no observed effect on victim‐reported abuse. While the former measure could be indicative of changes in victim willingness to call the police, the latter may be a more accurate measure of abuse prevalence (given that police reports may be partially dependent on victims' confidence in the police). A rapid literature review by Mazerolle et al. (2018, p. 30) concluded that:

… perceptions of and experiences with police directly influence victim's willingness to report. A perceived inadequate response by police in the past …. can make victims reluctant to report re‐victimisation.

In other words, due to low reporting rates at baseline (Felson & Paré, 2005; Pate et al., 1992; Strong & Cohen, 2013), prior studies have suggested that changes in official data estimates of repeat abuse may best represent changes in victim willingness to contact police (see Davis & Taylor, 1997; Davis et al., 2008; Hovell et al., 2006). Conversely, measures of repeat victimization captured through victim surveys and interviews may be less subject to reporting error, perhaps leading to more accurate estimates of repeat victimization (Casey et al., 2007; Greenspan et al., 2005). Any finding of significant changes to police‐reported repeat abuse may be misinterpreted unless viewed alongside comparable measures of victim‐reported abuse, and any discrepancies between these two measures may help to distinguish between results that are attributable to reporting effects and those that are attributable to behavioral effects. Thus, one interpretation of the original review's findings might be that second responder interventions increase victim confidence in the police but do not actually reduce the true prevalence of repeat abuse.^1^

Unfortunately, these results do not give a clear indication as to whether this type of intervention merits public funding. Nonetheless, the second responder model has continued to be adopted by police agencies in the United States, with New York City recently expanding a pilot program in five precincts to all 76 NYPD precincts.^2^ Second responder programs have also been implemented in other countries. Meyer (2011) reports on the creation of the PRADO (Partnership Responses at Domestic Violence Occurrence) program in Australia, now expanded to several additional sites. Evaluations of second responder programs have also been conducted in Canada (Scott et al., 2015) and the United Kingdom (Koppensteiner et al., 2019). Thus, given the inconclusiveness of the original review and the evidence of increased program implementation and evaluation, an updated review is warranted.

Finally, second responder interventions vary on numerous aspects related to treatment delivery and implementation. Given that these programs seek to capitalize on a small window of opportunity with which to intervene with victims of family abuse (Anderson et al., 1995; Davis & Smith, 1994), it seems plausible that the amount of time that elapses between a family abuse complaint and the second response may impact the efficacy of the intervention. Some have further suggested that the intention of these programs is to create long‐term solutions to family abuse, which may take considerable time to materialize (Davis & Taylor, 1997). If so, long‐term follow‐up periods may be needed to properly test for desired treatment effects. Unfortunately, research efforts frequently suffer from low face‐to‐face contact and survey retention rates (Davis et al., 2001; Koppensteiner et al., 2019; Stover et al., 2009; Stover et al., 2010), and it is currently unclear whether these difficulties further obscure the effect of the intervention on those who do receive it.

Second responder evaluations also differ in the units of analysis that they follow. While some studies measure police‐reported repeat abuse using households or families (Davis & Taylor, 1997; Hovell et al., 2006), others follow individual victims or victim/offender pairings (Davis et al., 2010; Pate et al., 1992). If victims are able to successfully leave an abusive partner or household during the study period, however, the unit of analysis may become important. Certain measures may be more or less sensitive to victim/offender separation, or even victimization committed by a new intimate partner.

Considering that the impact of these interventions may be tied to their ability to contact victims quickly and successfully, or even to encourage victims to access services and leave their abusers, characteristics such as these may be important determinants of treatment effects. As such, this updated review also provides a much needed exploration into the moderators and mechanisms associated with second responder interventions.

## 3 OBJECTIVES

This review seeks to extend the results of the prior review by providing an updated synthesis of the published and unpublished empirical research on the efficacy of second responder interventions for reducing repeat family abuse. Specifically, this review focuses on the following research questions:

- What impacts do second responder programs have on officially reported incidents of repeat family abuse?
- What impacts do second responder programs have on victim reported incidents of repeat family abuse?

Part of the logic model of second responder interventions involves the provision and use of victim services as an intermediate mechanism through which repeat abuse may be prevented (Mazerolle et al., 2018). As such, we also extend the objectives of the original review by examining the impact of second responder interventions on the use of victim services following the intervention.

A final objective of this review is to examine whether the effects of second responder programs appear dependent on a number of potential moderating characteristics, such as the research design, the speed of the second response, the length of the follow‐up period, the survey retention/face‐to‐face contact rate, and the unit of analysis evaluated. The hope is that these findings can shed further light on the specific aspects of second responder interventions that influence their success, and to extend both policy and research implications beyond the simple determination of whether these programs appear to work, on average.

## 4 METHODS

### 4.1 Criteria for considering studies for this review

#### 4.1.1 Types of studies

The scope of this review was limited to experimental and nonequivalent control group quasi‐experimental designs (see Cook & Campbell, 1979; Shadish et al., 2002). Eligible studies were required to include a comparison group of family abuse incidents that did not receive a second response intervention. Eligible studies were also required to report on at least one measure of repeat family abuse taken during the post‐intervention period. These measures could have included intimate partner violence, elder abuse, or family abuse more generally.

Comparison groups were considered eligible if they consisted of family abuse incidents that met the same criteria as treatment group incidents (i.e., intimate partner violence complaints in both groups, elder abuse complaints in both groups, general family abuse complaints in both groups, etc.). Comparison groups could be drawn from either the same geographic area (e.g., police precinct) as the treatment group, from another geographic area identified as having a similar case and demographic make‐up, or from an earlier (pre‐intervention) time period.^3^ Matching of treatment and comparison groups was not required for this review, however, quasi‐experimental studies that did not match treatment and comparison groups were required to demonstrate some effort to limit the influence of confounding factors. That is, quasi‐experimental studies with unmatched groups were only included if the comparison group had either face validity or covariates were included in the outcome analysis (e.g., multiple regression with covariates, ANCOVA, etc.), in which case, effect sizes were taken from these covariate‐adjusted estimates. Eligible studies were not required to report pre‐intervention measures of victimization.

More broadly, methodological requirements followed those of the Global Policing Database (GPD; www.gpd.uq.edu.au). The following list of eligible study designs was adapted from the GPD protocol (see Higginson et al., 2015, pp. 47–48):

- Randomized controlled trials (RCTs)/experimental designs.
- Regression discontinuity designs.
- Matched control group designs with or without pre‐intervention baseline outcome measures (statistical or propensity score matching).
- Matched control group designs with pre‐intervention measures of descriptive statistics (comparison of baseline characteristics).
- Unmatched control group designs where the control group has face validity.
- Unmatched control group designs analyzed with covariates.

#### 4.1.2 Types of participants

Second responder interventions aim to address repeat abuse that occurs within domestic and intimate partner relationships through direct contact with the victim. However, at times, these interventions may also attempt to address the behaviors of the offender and the household more generally. As such, our populations of interest included all victims, offenders, and households experiencing repeat family abuse (e.g., assault, battery, threats, harassment, etc.). These populations are not limited to any demographic, geographic, and socioeconomic characteristics.

#### 4.1.3 Types of interventions

This review defines second responder interventions as any program operated by or in cooperation with a municipal law enforcement agency where, in response to a family abuse complaint (complaints involving intimate partners, family members, or persons cohabiting), the police summon a family abuse specialist or specialists to visit victims at their homes or a police station. These specialists could be victim advocates and/or specially trained police officers. For interventions to be eligible, we required the nature of this contact to be aimed at addressing repeat abuse and connecting the victim with services. This could include the provision of information about the nature of family abuse, safety planning, legal rights, shelter placement, relocation assistance, and referrals to various services (e.g., domestic violence services, mental health services, court advocacy services, etc.). Lastly, the intervention was required to involve some level of face‐to‐face contact between the victim and the second responder(s), even if this contact was only successfully provided to a subset of eligible victims. Interventions that consisted entirely of mail or phone contact were excluded.

#### 4.1.4 Types of outcome measures

##### 4.1.4.1 Primary outcomes

This review focused on two main outcome measures:

- Repeat family abuse/victimization as measured by official data sources.
- Repeat family abuse/victimization as measured by unofficial data sources.

Borrowing from the Center for Disease Control and Prevention's (CDC) definition of intimate partner violence, here we define family abuse as any incident of abuse or aggression involving family or household members, which could include physical violence, sexual violence, stalking, and/or psychological aggression.^4^ In addition, eligible incidents were required to fit under one of the following conditions:

- Repeat incidents committed against the same victim and by the same offender.
- Repeat incidents committed against the same victim by any offender.
- Repeat incidents committed by the same offender against any victim.
- Repeat incidents occurring within the same household.

Taken together, eligible outcomes involved any incident of assault, battery, threats, stalking, and/or harassment, etc. that occurred between individuals engaged in an intimate relationship and/or residing in the same household. Official data sources included calls for service, arrests, and crime incident reports, and unofficial data sources included victim surveys and interviews.

##### 4.1.4.2 Secondary outcomes

One of the primary mechanisms through which second responder programs are proposed to reduce repeat incidents of family abuse is through increased provision of victim services (Mazerolle et al., 2018). While not initially anticipated, a sufficient number of eligible studies reported post‐intervention service use as an outcome and provided data from which an effect size could be calculated. Thus, we also examined the impact of second responder programs on the use of victim services as a secondary outcome. These services were limited to those not provided by a police organization, such as support groups, shelters, mental health services, and court services, etc.

#### 4.1.5 Duration of follow‐up

Eligible studies were not limited to any specific follow‐up periods. While second responder interventions are intended to capitalize on a small window of opportunity after a family abuse incident occurs, the anticipated effects of the intervention are both short‐term and long‐term. In the short‐term, these responses may increase immediate service uptake and willingness to report victimization to the police. However, reducing the prevalence of repeat family abuse may be a more long‐term process, particularly given the complicated attachments that often exist between victims and abusers (see Davis & Taylor, 1997; Frank & Golden, 1992; Hovell, 2006). As such, the duration of follow‐up across our eligible studies displayed significant variability, ranging from approximately several months or less (Stover et al., 2010) to multiple years (Friday et al., 2006; Mizrachi, 2019).

#### 4.1.6 Types of settings

No restrictions were placed on study setting. The units of analysis included in our eligible studies were drawn from a variety of geographic, cultural, demographic, and ethnic contexts. This included studies conducted in various cities and countries, and with samples comprised of various demographic and socioeconomic characteristics.

### 4.2 Search methods for identification of studies

#### 4.2.1 Electronic searches

The primary search strategy for this review was conducted by the Global Policing Database (GPD) research team at the University of Queensland (Elizabeth Eggins and Lorraine Mazerolle) and Queensland University of Technology (Angela Higginson). The GPD is described as a web‐based and searchable database designed to capture all published and unpublished experimental and quasi‐experimental evaluations of policing interventions conducted since 1950 (Higginson et al., 2015). There are no restrictions on the type of policing technique, type of outcome measure or language of the research. The GPD is compiled using systematic search and screening techniques, which are reported in Higginson et al. (2015) and summarized in Supporting Information Appendices B and C. Broadly, the GPD search protocol includes an extensive range of search locations to ensure that both published and unpublished research is captured across criminology and allied disciplines. Full GPD functionality is not publicly available, however, and complete searches must be conducted by the GPD team.

The following keyword strings were used to search the title and abstract fields of the GPD corpus of full‐text documents that have been screened as reporting on a quantitative impact evaluation of a policing intervention and using an eligible research methodology (as described above). Here, “TI” represents keyword combinations that were limited to title fields and “AB” represents keyword combinations that were limited to abstract fields. As these searches were meant to update those of the original review, search results were limited to studies published from 2004 to December 2019:

1. (TI: ("second respon*" OR coordinate* OR multiagency OR integrated OR visit* OR service* OR interven* OR program* OR advocate* OR "social work*" OR counsel* OR psychologist* OR health* OR clinician*)) OR (AB: ("second respon*" OR coordinate* OR multiagency OR integrated OR visit* OR service* OR interven* OR program* OR advocate* OR "social work*" OR counsel* OR psychologist* OR health* OR clinician*))
  AND
2. (TI: (domestic* OR wife OR wives OR husband* OR partner* OR intimate* OR relationship* OR family OR familial OR families)) OR (AB: (domestic* OR wife OR wives OR husband* OR partner* OR intimate* OR relationship* OR family OR familial OR families))
  AND
3. (TI: (abuse* OR aggress* OR assault* OR batter* OR coercive* OR chok* OR death* OR beat* OR harm* OR femicide* OR homicid* OR infanticide OR lethal* OR murder* OR manslaughter* OR injur* OR shoot* OR stab OR stabb* OR strangl* OR strangul* OR violen* OR weapon*)) OR (AB: (abuse* OR aggress* OR assault* OR batter* OR coercive* OR chok* OR death* OR beat* OR harm* OR femicide* OR homicid* OR infanticide OR lethal* OR murder* OR manslaughter* OR injur* OR shoot* OR stab OR stabb* OR strangl* OR strangul* OR violen* OR weapon*))

We also extended the GPD search by reviewing research and publications reported in the following gray literature repositories. These repositories dedicate specific focus to issues of domestic violence in contexts outside of the United States:

- Queensland Center for Domestic and Family Violence Resources (https://noviolence.org.au/)
- Australia's National Research Organization for Women's Safety (https://www.anrows.org.au/)
- Stopping Family Violence (https://sfv.org.au/)
- Domestic Violence Prevention Centre (https://domesticviolence.com.au/)
- SaveLives (https://safelives.org.uk/about-us)
- Women's Aid (https://www.womensaid.org.uk/)
- Women Against Violence Europe (https://www.wave-network.org/)
- The Federal Ministry for Family Affairs, Senior Citizens, Women and Youth (https://www.bundesregierung.de/breg-en/federal-government/ministries/ministry-of-family-affairs)

#### 4.2.2 Searching other resources

Several additional strategies were used to supplement our electronic searches. First, we searched 2020 and 2021 issues of leading academic journals through October of 2021 to identify any recent studies that were not yet indexed in the GPD.^5^ Second, we reviewed the reference lists of relevant prior reviews that were identified during our GPD searches.^6^ Third, we searched the U.S. Office of Violence Against Women (https://www.justice.gov/ovw) website for a listing of federally funded second responder programs and any evaluations conducted on those programs. Finally, we examined the reference lists of all eligible studies and emailed our final eligibility list to the authors of these studies. This was done in attempt to identify additional research that our search strategies had not detected, particularly dissertations and other unpublished reports.

### 4.3 Data collection and analysis

#### 4.3.1 Selection of studies

Identified studies first underwent title and abstract screening by one of the authors of this review (Petersen or Davis). To establish consistency, a pilot sample of 25 abstracts was assigned to both screeners, who were asked to determine if the results were potentially relevant or were clearly not relevant. Results of these pilot screenings were then compared and discussed, which indicated a high level of agreement between screeners (96%). The remaining titles and abstracts were then divided between screeners and independently coded as being either potentially relevant or not relevant. Any results that generated uncertainty were discussed among the review authors. All titles and abstracts were screened using *Abstrackr* (Wallace et al., 2012), which is a free online service designed for abstract screening in systematic reviews.

We then retrieved full‐text reports for all results deemed potentially relevant at the title/abstract stage. Full‐text reports were once again divided amongst screeners (Petersen and Davis) and were coded as being either eligible for inclusion, ineligible for inclusion, or “on the fence.” For ineligible studies, we also coded information concerning the reasoning for ineligibility (e.g., duplicate result, ineligible outcomes, ineligible intervention, or ineligible methodology). Any studies identified as “on the fence” were reviewed and discussed among all authors of this review, and consensus was achieved on all eligibility decisions. Lastly, any prior reviews that were ineligible but appeared relevant for potential reference harvesting were labeled as “relevant for review.”

#### 4.3.2 Data extraction and management

All studies identified as eligible for inclusion were double coded by Kevin Petersen (the first author of this review) and an additional research assistant at George Mason University. Our coding protocol (Supporting Information Appendix D) consisted of a variety of items including (but not limited to):

- Reference information (publication type, research team, etc.).
- Description of the study site and intervention (geographic area, nature of the second response, dosage and intensity, etc.).
- Methodology (assignment process, nature of treatment and control groups, sample sizes, etc.).
- Implementation issues (risk of bias, threats to internal validity, etc.).
- Results (raw data, statistical significance, etc.)
- Conclusions reached by the authors.

Data was entered and stored using *EpiData Software* (https://www.epidata.dk/index.htm) and extracted to.csv files for analysis. Data validation was also performed to compare coding results between coders. Any coding discrepancies were discussed among study authors, where a final coding decision was reached.

#### 4.3.3 Assessment of risk of bias in included studies

For each eligible study, risk of bias measures were independently coded using items taken from the Cochrane randomized and non‐randomized risk of bias tools (Sterne et al., 2016; Sterne et al., 2019). Rather than apply the full assessment tools for each eligible study, we used a combination of slightly modified items from each instrument, which were determined to be directly relevant to our body of research (see Table 4). This allowed us to classify all studies on a common scale, ranging between low risk of bias, some concerns, or high risk of bias.

#### 4.3.4 Measures of treatment effect

Based on the prior review (Davis et al., 2008), and as discussed in our protocol (see Davis et al., 2021), we anticipated that the average number of repeat family abuse incidents per victim, household, or offender during a typical follow‐up period would be low. Consequently, we expected variation in the way that outcome measures would be reported across studies, with some studies likely reporting incidence data (e.g., mean number of repeat incidents) and others reporting prevalence data (e.g., proportion or frequency of victims who experience a repeat incident). Upon coding our eligible studies, it became evident that this was the case, and thus we calculated Hedge's *g* effect sizes when repeat victimization was operationalized as a continuous outcome and logged odds ratios when repeat victimization was operationalized as a dichotomous outcome (see Borenstein et al., 2009).

Given the noncomparability of standardized mean differences and odds ratios, however, conversions were needed to present all effect sizes in a common metric. While several methods exist for converting standardized mean differences to odds ratios (and vice versa), these methods are only approximations and do not necessarily provide the exact effect that would have been observed had the outcome been scaled or dichotomized (Weisburd et al., 2022). Given this consideration, our intention was to limit the number of conversions that would be needed to present all effect sizes in a common metric. After completing our screening and coding processes, it was determined that the vast majority of eligible studies measured repeat family abuse dichotomously, and thus logged odds ratios were used as our primary effect size metric.

Dichotomous measures of repeat abuse were most commonly reported using binary proportions. For these studies, we used the proportions and the sample sizes to calculate cell frequencies as follows:

$$\begin{array}{l} a=p1\times n1\\ b=n1-a\\ c=p2\times n2\\ d=n2-c \end{array}$$

where *p*1 represents the proportion of cases in the treatment group that experienced a repeat family abuse incident, *n*1 represents the sample size of the treatment group, *p*2 represents the proportion of cases in the control group that experienced a repeat family abuse incident, and *n*2 represents the sample size of the control group. We then used these cell frequencies to compute the natural log of the odds ratio and the variance of the logged odds ratio as shown below (see Lipsey & Wilson, 2001):

$$\begin{array}{l} \ln\left(OR\right)=\ln\left(\frac{ad}{bc}\right)\;\\ V\ln\left(OR\right)=\;\frac{1}{a}+\frac{1}{b}+\frac{1}{c}+\frac{1}{d} \end{array}$$

where *a* is the number of cases in the treatment group that experienced a repeat family abuse incident, *b* is the number of cases in the treatment group that did not experience a repeat family abuse incident, *c* is the number of cases in the control group that experienced a repeat family abuse incident, and *d* is the number of cases in the control group that did not experience a repeat family abuse incident. Several studies provided estimates from logistic regression models rather than raw proportions or frequencies. Here we used the logged odds ratios and standard errors directly reported by these models. We then squared the reported standard error to calculate the effect size variance. At times, it was necessary to calculate the standard error and variance from 95% confidence intervals. Since the threshold for statistical significance based on the standard normal distribution is 1.96 standard errors (*p* < 0.05), we divided the logged confidence interval by two times this significance threshold (see Higgins et al., 2019):

$$SE=\frac{{\mathrm{CI}}_{\mathrm{Upper}}-{\mathrm{CI}}_{\mathrm{Lower}}}{3.92}$$

When necessary, Hedge's *g* effect sizes were calculated using the means, standard deviations, and sample sizes of the treatment and control groups. Only on rare occasions were alternative methods necessary. For instance, for the Regoeczi and Hubbard (2018) study, a standardized effect size was calculated using the results of a *χ* ^2^ test and the overall sample size of the test. Conversion of Hedge's *g* effect sizes to logged odds ratios for our main outcome measures was done using the Cox logit method, which multiplies the standardized mean difference by 1.65 and divides the variance of the mean difference by 0.367 (see Sánchez‐Meca et al., 2003). The formulas and functions for all effect size calculations are described in Supporting Information Appendix E.

As previously noted, we also examined victim use of services during the post‐intervention period as a secondary outcome. Studies reporting this outcome were evenly split between prevalence and incidence data. As such, we estimated models using both Hedge's *g* and logged odds ratios for this outcome. Conversions for both metrics were once again conducted using the Cox logit method. All calculations were done using manual functions built in R statistical software (R Core Team, 2020) as well as David Wilson's effect size calculator (https://www.campbellcollaboration.org/escalc/html/EffectSizeCalculator-Home.php).

#### 4.3.5 Criteria for determination of independent findings

Our unit of analysis for this review is the research study (defined here as each unique research sample). To maintain independence, each study/sample was included no more than once in any given analysis. Several studies were associated with multiple research reports; however, these reports were used only to increase the comprehensiveness of the study coding process.

At times, individual studies also reported multiple relevant outcomes from within the same general outcome grouping (i.e., repeat family abuse based on official data sources, repeat family abuse based on unofficial data sources, victim use of services). This situation presents concern over the potential “creaming” (Braga et al., 2019, p. 7) of effect sizes, or the intentional selection of effects that are either favorable or unfavorable to treatment. To address these issues, we followed a number of a priori selection rules laid out in our initial protocol. First, studies generally provided aggregate or general measures of repeat family abuse, which we prioritized over more specific measures. Second, we prioritized measures of repeat family abuse that occurred within the same victim/offender pairings or households over measures that followed the same victim or offender alone. Similarly, we prioritized measures of repeat family abuse following the same victim over those following only the same offender. We also prioritized adjusted effects sizes over unadjusted effect sizes, when possible. For example, Pate et al. (1992) provide raw prevalence data as well as estimates from logistic regression models that included baseline covariates. We used the effect sizes from the regression models here, given that these models account for factors that may confound the treatment effect. Lastly, we prioritized intent‐to‐treat effect sizes over effect sizes related to treated samples only. Several studies provided estimates of both, and we conducted sensitivity analyses using the treated‐only effects for these studies.

The above strategies were generally successful at reducing our outcome constructs to one eligible effect per study. However, two studies reported multiple outcomes that could not be addressed through the above selection rules. Stover et al. (2009) reported separate treatment effects across varying levels of engagement with the second response intervention. Similarly, Stover et al. (2010) reported victim service usage in the immediate aftermath of the intervention separately for different types of service. Given the rarity of this situation, the effect sizes and variances reported in these two studies were simply averaged at the study‐level. This approach has been used in recent systematic reviews related to policing interventions and ensures that each study remains represented by only one effect size (see Lum et al., 2020).

A related issue concerns the reporting of multiple follow‐up periods. While the majority of our eligible studies contained only one follow‐up period from which an effect size could be calculated, two studies reported multiple follow‐up periods with corresponding outcome data. To handle these studies, we prioritized the longest follow‐up period as this is most analogous to a combined or aggregate effect size and is most consistent with the recommendations for research on domestic abuse (see Sherman et al., 1992). However, we also estimated models using the shortest follow‐up periods for these studies as a sensitivity analysis.

As a final note, the initial protocol for this review proposed the use of either separate models or robust variance estimation (see Hedges et al., 2010) if a significant number of studies reported multiple conceptually similar outcomes that could not be reduced via the above selection rules (e.g., multiple forms of repeat abuse using the same data source). Ultimately, these situations did not arise with enough frequency to allow for the meaningful estimation of separate models, and there was little conceptual overlap in the studies that did report multiple outcomes. For instance, only four studies reported multiple forms of repeat abuse using the same data source (Davis et al., 2001; Davis et al., 2010; Greenspan et al., 2005; Pate et al., 1992), which included measures of both physical and/or psychological abuse. There was very little clear overlap in the specific data sources and types of sub‐outcomes reported in these studies, however, with some studies measuring physical or psychological abuse broadly and others measuring specific events such as threats or property damage. In addition, the use of robust variance estimation would have provided little additional value, given that each of these studies also reported an aggregate measure of repeat abuse which inherently combined these more specific outcomes into a single effect. In a similar fashion, our eligible studies did not provide separate estimates for different forms of family abuse complaints (e.g., intimate partner violence, elder abuse, etc.). To explore the potential for treatment effects to differ by complaint type, we were forced to examine this variable in a categorical moderator analysis.

#### 4.3.6 Dealing with missing data

When studies that were otherwise eligible did not report the requisite data to calculate an appropriate effect size, we attempted to contact study authors. Ultimately, there were no eligible studies from which we were unable to calculate at least one effect size.

#### 4.3.7 Assessment of heterogeneity

Heterogeneity of effect sizes was assessed using the *Q, τ* ^2^, and *I* ^2^ statistics. The *Q* statistic was used to test for excess heterogeneity (more than would be expected due to sampling error alone), and whether excess heterogeneity was statistically significant. The *τ* ^2^ statistic was used to represent the degree of variance added to the analysis through random effects estimation (if applicable; see Borenstein et al., 2010), and the *I* ^2^ statistic was used to represent the proportion of the total variability in effect sizes that could be attributed to heterogeneity between studies. *I* ^2^ values of less than 30% were considered mild, values from approximately 30‐60% were considered moderate, and values greater than 60% were considered more substantial (see Higgins & Thompson, 2002).

#### 4.3.8 Assessment of reporting biases

We used four approaches to test for potential reporting bias. The first was a simple moderator analysis testing for significant differences in effect sizes between published and unpublished reports. We then viewed funnel plots for each of our outcome models, looking for any visual signs of asymmetry associated with larger or smaller standard errors. Third, we conducted trim‐and‐fill analyses for each funnel plot (Duval & Tweedie, 2000). If significant asymmetry was detected, we then examined any changes in the mean effect size that occurred due to the imputation of additional effect sizes by the trim‐and‐fill analysis. Finally, we conducted Egger's regression tests for each of our outcome models. Conceptually, this test regresses the effect sizes on their standard errors to determine if there is a linear relationship between the size of the observed effects and the precision of their estimates (Egger et al., 1997).

#### 4.3.9 Data synthesis

Random effects models with restricted maximum likelihood estimation were conducted using the *metafor* package (Viechtbauer, 2010) in R statistical software (R Core Team, 2020). Given that our studies involved various techniques occurring across various contexts and settings, we felt it unlikely that these interventions would share a common effect. Furthermore, the use of random effects models is now considered best practice, as the results of these models will converge on the fixed‐effects model in the absence of excess heterogeneity (Borenstein et al., 2010). For outcomes that are reported using odds ratios, we estimated these models using the natural log of the odds ratio. However, our final results were exponentiated to represent the percentage point difference in the odds of experiencing a repeat incident of family abuse for treatment groups relative to control groups.

#### 4.3.10 Subgroup analysis and investigation of heterogeneity

To investigate potential sources of heterogeneity across study results, we conducted a number of different moderator analyses. Categorical moderators were analyzed using the analog to the analysis of variance (ANOVA) method (see Lipsey & Wilson, 2001) and continuous moderators were analyzed using meta‐regression (see Higgins et al., 2020).^7^ As discussed in the initial protocol, these moderators included:

- Research design (experimental vs. quasi‐experimental).
- Length of time between triggering incident and second response (immediate or within 72 h, within 7 days, more than 7 days).
- Type of family abuse complaint (intimate partner abuse vs. general family abuse or elder abuse).
- Length of data collection follow‐up period (number of months that repeat abuse is tracked).
- Type of report (published vs. unpublished).

Upon screening and coding our eligible studies, we also identified a number of additional variables that could impact the effects of the intervention and were reported with enough consistency to employ as moderators. These included:

- Face to face contact rate of the intervention.
- Survey/interview retention rate (for unofficial data measures).
- Incidents eligible for the intervention (complaints resulting in arrest vs. any complaint, founded or unfounded).
- Confounding interventions (was the intervention limited to a second response or did it include additional components?).
- Unit of analysis (same victims, same victim/offender pairing, same household).

As previously noted, the above moderators may be particularly important. Successfully locating and contacting victims to conduct second responses and retaining victim cooperation for follow‐up surveys and interviews are both major challenges for second responder programs (see Davis & Medina, 2001; Davis et al., 2010). Victims who cannot be contacted may be at greater risk of experiencing repeat abuse, while victims who remain in the study during the follow‐up period may be more likely to access services and other support mechanisms. Research on domestic violence interventions has also suggested that the use of arrest has significant and variable effects on recidivism (see Sherman, 2018; Sherman et al., 1992). Accordingly, the impacts of second responder interventions may vary depending on whether the offender is arrested following the triggering incident.

Second responder evaluations also vary in the unit of analysis that they follow, with some limiting their measures of repeat family abuse to the same victim/offender pairings or households (Davis et al., 2010), and others following the same victim or offender more generally (Mizrachi, 2019; Regoeczi & Hubbard, 2018). Given that these interventions may empower victims to leave their abusers, the unit of analysis could have important implications for study findings.^8^ Lastly, several studies evaluated programs with multiple components, but these components could not be separated into discrete effects. To determine whether these confounding interventions could have impacted our findings, we compared the mean effect sizes for these studies with those of studies that involved only the second response.

#### 4.3.11 Sensitivity analysis

In addition to the moderators listed above, we conducted sensitivity analyses based on follow‐up duration and the level of treatment. Specifically, two studies (Hovell et al., 2006; Koppensteiner et al., 2019) provided data that allowed us to calculate effect sizes for both a shortest and a longest follow‐up duration (for official measures of repeat abuse only). Additionally, three studies provided data that allowed us to calculate effect sizes for different levels of treatment. Two of these studies (Friday et al., 2006; Stover et al., 2009) reported estimates of repeat family abuse for different levels of engagement with the intervention, and one study (Messing et al., 2015) provided data on the use of victim services for both an intent‐to‐treat and a treated group.^9^ While we prioritized the longest follow‐up period and the intent—to‐treat estimates in our primary models, we also estimated these models using the shortest follow‐up period the treatment‐only estimates, where appropriate.

### 4.4 Deviations from protocol

There are several deviations from the protocol for this review that deserve mention (see Davis et al., 2021). In the initial protocol, we did not discuss the use of victim services as a secondary outcome. This is because we did not originally anticipate that this measure would be reported in a sufficient number of studies to allow for any meaningful analysis. It should be noted here that we also did not specifically search or screen for the use of victim services, and all eligible studies were still required to report a measure of repeat family abuse. Thus, this outcome measure should be considered strictly secondary.

We have also expanded our list of moderator variables from that which was originally proposed, namely through the addition of face‐to‐face contact rate, survey retention rate, the type of incidents eligible for the second response, the unit of analysis, and whether there were confounding interventions. Again, this was due to uncertainty regarding the information that would be consistently reported in our eligible studies, though our initial protocol did note that additional post‐hoc moderator variables may be added depending on the characteristics of our studies. Similarly, our initial protocol proposed the use of sampling method (volunteer vs. non‐volunteer) as an effect size moderator. This has been removed here due to a lack of variation in these methods. That is, all studies reporting official data measures used non‐volunteer methods to assign incidents to a second response, while all unofficial data measures inevitably involved volunteers.^10^

Finally, we added and/or adapted several coding items (Supporting Information Appendix D). We changed the categories used to describe the speed of the second response to “Immediate or within 72 h,” “Within several days of the incident (7 or less),” “More than several days after the incident (more than 7),” and “Other” to better fit the characteristics of our eligible studies. We also added a yes or no question to capture whether the second response could have been confounded by any additional interventions and an additional question inquiring about the unit of analysis used in the study.

## 5 RESULTS

### 5.1 Description of studies

#### 5.1.1 Results of the search

**Figure 1:**
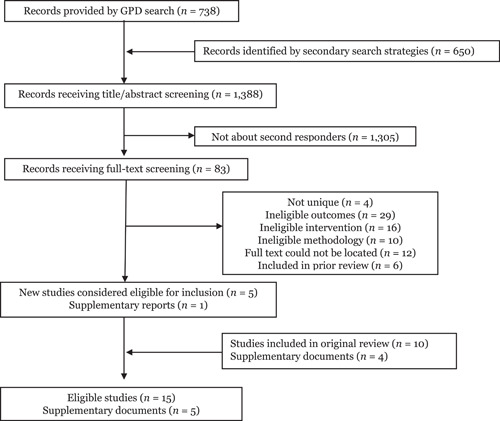
PRISMA flowchart for search results

The results of our various search strategies are reported in the PRISMA diagram seen in Figure 1. The GPD search conducted between 2004 and 2019 yielded 738 results. We then collected another 650 results through secondary strategies such as gray literature searches, reference harvesting from prior reviews, and searching 2020 and 2021 volumes of leading academic journals. After screening out abstracts that clearly did not involve second responder programs, we identified 83 potentially relevant reports, of which, 5 unique studies (and 1 supplementary document) were deemed eligible for inclusion. In combination with the prior review, this produced a population of 15 eligible studies associated with 20 separate documents. Of note, several unpublished reports were included in the original review and have since been published. Some of these studies were identified by our updated search strategies. while we do not consider these to be newly eligible studies, we used these more recent reports for coding purposes.

Many of our exclusion decisions could be easily made based on the outcomes reported by the study and/or the methodology that the study used. However, decisions regarding the eligibility of the intervention were often more difficult. A frequent issue was determining whether an intervention involved or attempted to involve any form of face‐to‐face contact with the victims. When we were uncertain about this, but studies were otherwise eligible, we attempted to contact the study authors for clarity. This strategy successfully produced consensus on several studies that initially generated debate (e.g., Friday et al., 2006; Regoeczi & Hubbard, 2018; Weisz et al., 2006). Also of note, in two of our eligible studies (Messing et al., 2015; Mizrachi, 2019), the second response initially appeared to be limited to phone contact. However, the initial phone call took place during face‐to‐face contact with a police officer, and subsequent face‐to‐face meetings were encouraged. Thus, we considered these studies to fit our eligibility criteria and the spirit of second responder programs more generally.

#### 5.1.2 Included studies

We did not encounter any issues that prevented us from calculating at least one effect size for each eligible study. As such, all 15 studies (10 from the prior review and 5 from the updated searches) were included in our analyses. The labels used for each study can be seen in Table 1, along with all associated documents. Of note, several documents were associated with multiple studies (Davis and Taylor, 1997; Davis & Maxwell, 2002; Davis & Medina, 2001; Davis et al., 2001; Davis et al., 2006). These reports all concern a trio of experiments evaluating the same domestic violence intervention education project (DVEIP) developed in New York City (NY) between 1987 and 1997. Davis and Taylor (1997) studied the effect of the program on a combined sample of incidents involving intimate partner violence, children abusing parents, and more general forms of family abuse. Davis et al. (2001) and Taylor (n.d.) evaluated the same intervention, but the former used a restricted sampling frame consisting of only elder abuse complaints, while the latter predominately involved intimate partner violence complaints. Thus, all three studies evaluated the same general program using an experimental design, but each used an independent sample of incidents. Pooled analysis of these studies was then conducted by Davis and Maxwell (2002) and Davis et al. (2006). As in the prior review, we chose to treat each experiment as an independent study rather than use the pooled analysis. However, prevalence data analyzed and reported in the pooled analysis was needed to calculate logged odds ratios for these three studies.

**Table 1:** Eligible studies (*N* = 15) and associated documents

Study label	Associated documents
Casey et al. (2007)	Casey, Berkman, Stover, Gill, Durso, and Marans (2007)
Davis and Taylor (1997)	Davis and Maxwell (2002); Davis, Maxwell, and Taylor (2006)
Davis et al. (2001)	Davis, Medina, and Avitabile (2001); Davis and Medina (2001); Davis and Maxwell (2002); Davis, Maxwell, and Taylor (2006)
Davis et al. (2010)	Davis, Weisburd, and Hamilton (2007); Davis, Weisburd, and Hamilton (2010)
Friday et al. (2006)	Friday, Lord, Exum, and Hartman (2006); Exum, Hartman, Friday, and Lord (2010)
Greenspan et al. (2005)	Greenspan, Weisburd, Lane, Ready, and Crossen‐Powell (2005)
Hovell et al. (2006)	Hovell, Seid, and Liles (2006)
Koppensteiner et al. (2019)	Koppensteiner, Matheson, and Plugor (2019)
Messing et al. (2015)	Messing, Campbell, Brown, Patchell, and Wilson (2015)
Mizrachi (2019)	Mizrachi (2019)
Pate et al. (1992)	Pate et al. (1992)
Regoeczi and Hubbard (2018)	Regoeczi and Hubbard (2018)
Stover et al. (2009)	Stover, Poole, and Marans (2009)
Stover et al. (2010)	Stover, Berkman, Desai, and Marans (2010)
Taylor (n.d.)	Taylor (n.d.); Davis and Maxwell (2002); Davis, Maxwell, and Taylor (2006)

There was a similar relationship between the studies conducted by Casey et al. (2007), Stover et al. (2009), and Stover et al. (2010). Each of these evaluates the domestic violence home‐visit intervention program (DVHVI) that began operation in New Haven (CT) during the early 2000s. Both the Casey et al. (2007) and Stover et al. (2009) studies used official data measures of repeat family abuse, however, the Casey et al. (2007) study was an earlier pilot test involving a separate group of incidents. There was overlap, however, between the Stover et al. (2010) and Stover et al. (2009) studies, where the latter analyzed a subset of the sample used in the former. However, Stover et al. (2010) reported only on unofficial data measures while Stover et al. (2009) reported on official data measures. Thus, each of these studies remains statistically independent in their respective analyses and no sample was included in any single model more than once.

Select characteristics for each eligible study are reported in Table 2. Nearly all eligible studies (*n* = 14, 93.3%) were conducted in the United States, with only one (Koppensteiner et al., 2019) being conducted in the United Kingdom. Additionally, a large proportion of studies were conducted in either New York City or New Haven (*n* = 6, 40%), and the majority of studies were funded by the National Institute of Justice (*n* = 8, 53.3%). There was a fairly even split between academic journal articles (*n* = 8, 53.3%) and government reports, dissertations, or other unpublished articles (7, *n* = 46.7%), and between quasi‐experimental designs (*n* = 9, 60%) and experimental designs (*n* = 6, 40%). In total, 12 studies (80%) reported on an official measure of repeat abuse and 8 studies (53.3%) reported on an unofficial measure of repeat abuse. An additional five studies (33.3%) reported on the use of victim services as a secondary outcome (Table 3), though we were unable to calculate an effect size for one of these studies (Davis et al., 2001). Narrative summaries for each study are also presented in Supporting Information Appendix A.

**Table 2:** Select study characteristics

Study name	Location	Publication type	Research design	*N* (official data)^a^	*N* (unofficial data)^a^	Funding source
Casey et al. (2007)	New Haven (CT)	Journal article	Quasi‐experimental	204	N/A	None
Davis and Taylor (1997)	New York (NY)	Journal article	Experimental	428	308	National Institute of Justice
Davis and Medina (2001)	New York (NY)	Government report	Experimental	372	272	National Institute of Justice
Davis et al. (2010)	Redlands (CA)	Journal article	Experimental	300	125	National Institute of Justice
Friday et al. (2006)	Charlotte (NC)	Government report	Quasi‐experimental	704	N/A	National Institute of Justice
Greenspan et al. (2005)	Richmond (VA)	Government report	Quasi‐experimental	N/A	120	National Institute of Justice
Hovell et al. (2006)	San Diego (CA)	Journal article	Quasi‐experimental	803	N/A	None
Koppensteiner et al. (2019)	Leicestershire (UK)	Government report	Experimental	1015	N/A	Ministry of Justice and the Office of the Leicestershire Police and Crime Commissioner
Messing et al. (2015)	Oklahoma	Journal article	Quasi‐experimental	N/A	414	National Institute of Justice
Mizrachi (2019)	Las Vegas (NV)	Dissertation	Quasi‐experimental	954	N/A	None
Pate et al. (1992)	Miami (FL)	Government report	Experimental	907	381	National Institute of Justice
Regoeczi and Hubbard (2018)	Cleveland (OH)	Journal article	Quasi‐experimental	1279	N/A	None
Stover et al. (2009)	New Haven (CT)	Journal article	Quasi‐experimental	508	N/A	None
Stover et al. (2010)	New Haven (CT)	Journal article	Quasi‐experimental	N/A	107	Substance Abuse and Mental Health Administration
Taylor (n.d.)	New York (NY)	Unpublished report	Experimental	177	86	National Institute of Justice

^a^ Where possible, this reflects the sample size used in the analysis of interest.

**Table 3:** Collective study characteristics

Characteristic	*N*	%
Research design		
Experimental	6	40
Matched quasi‐experimental	2	13.3
Unmatched quasi‐experiment	7	46.7
Outcome		
Repeat abuse (official data)	12	80
Repeat abuse (unofficial data)	8	53.3
Use of victim services	5	33.3
Publication type		
Journal article	8	53.3
Government report	5	33.3
Dissertation	1	6.7
General unpublished report	1	6.7
Funding		
Yes	10	66.7
No	5	33.3
Unit of analysis		
Same pairing or household	7	46.7
Same victim	7	46.7
Same offender	1	6.7
Speed of second response		
Within 72 h	8	53.3
Within 7 days	3	20
More than 7 days	3	20
Unclear	1	6.7
Type of complaint		
Intimate partner violence only	10	66.7
Elder and/or any family abuse	5	33.3
Eligible incidents		
Arrests only	4	26.7
Any report, founded or unfounded	11	73.3
Length of follow‐up period		
6 months or less	5	33.3
12 months or less	3	20
More than 12 months	3	20
Multiple follow‐up periods	4	26.7
Confounding interventions		
None	9	60
Public education	2	13.3
Validated risk screening	2	13.3
Enhanced investigation/prosecution	2	13.3

Additional information for the full collection of studies can be seen in Table 3, where we present descriptive statistics for the majority of our moderator variables. Only one study used the same offender as the unit of analysis, and the remaining studies were evenly split between the same victim and either the same household or the same victim/offender pairing.^11^ Just over half of all studies evaluated an intervention that attempted to contact victims within 72 h of the triggering incident, and two‐thirds analyzed intimate partner violence complaints only. In most studies, any family abuse report was considered eligible to receive the intervention, whether the report was founded or unfounded. The duration of follow‐up varied considerably across studies, with one‐third of all studies using a follow‐up period of 6 months or less, one‐fifth using a follow‐up period of 12 months or less, one‐fifth using a follow‐up period of greater than 12 months, and approximately one‐fourth using multiple follow‐up periods. As previously noted, however, we were only able to calculate separate effect sizes for two of the four studies with multiple follow‐up periods (Hovell et al., 2006; Koppensteiner et al., 2019).

Two‐fifths of our studies included a confounding intervention, or an additional intervention above and beyond that of the second response. These confounding interventions involved domestic violence educational campaigns (Davis & Taylor, 1997; Davis et al., 2001), the use of validated risk assessment tools (Messing et al., 2015; Mizrachi, 2019), and increased investigation and/or prosecution of family abuse incidents (Friday et al., 2006; Regoeczi & Hubbard, 2018). In addition to the above characteristics, there were 10 studies (66.7%) from which we could derive estimates of face‐to‐face contact rate, and 8 (53.3%) studies that reported survey or interview retention rates. Face‐to‐face contact rates ranged from 24% to 100% (*M* = 78.98, *SD* = 27.26) and survey retention rates ranged from 21% to 76% (*M* = 52.67, *SD* = 20.46). It should be noted, however, that the face‐to‐face contact rates were inflated by several quasi‐experimental studies that included only cases that received treatment.

#### 5.1.3 Excluded studies

There were three studies that warranted further discussion during our full text review but were ultimately determined to be ineligible. One was excluded because the second response appeared to be limited to phone contact and several elements of the response also occurred in the control group (Tromboli, 2017). Another study clearly involved a second response intervention, but the intervention was targeted at domestic offenders rather than victims (Scott et al., 2015). Finally, we excluded one study after communication with the primary author suggested that the nature of the intervention did not meet our eligibility criteria (Weisz et al., 2006).

#### 5.1.4 Study implementation

While the majority of eligible studies did not report issues with program implementation, several studies did encounter difficulties in treatment delivery and evaluation. Davis and Taylor (1997) reported a 17% rate of experimental misassignment, with approximately 2% of cases assigned to control receiving home visits and 15% of cases assigned to treatment failing to receive a home visit. The contamination of control cases occurred as the result of staff overrides (or staff ignoring the randomization), while the failure to deliver treatment to experimental cases was due to an inability to locate the victims. Similar issues were noted in other studies (Davis et al., 2001, 2010; Koppensteiner et al., 2019; Stover et al., 2009). Davis et al. (2001) reported that three control cases were overridden by program staff, and only 50% of targeted households received the full intervention. Eight control cases were overridden in Davis et al.'s (2010) study, with 16% of experimental cases failing to receive the full treatment. Finally, Stover et al. (2015) found that just 52% of their sample engaged with second responders at any level, and only 25% of households in Koppensteiner et al.'s (2019) study received face‐to‐face contact (though an additional 40% were successfully contacted by phone). Once again, second responders in these studies often encountered issues successfully locating and contacting victims (see also Regoeczi & Hubbard, 2018). For Davis et al. (2001), these issues also caused significant delays to the delivery of the intervention, which was intended to be conducted within a few days but ultimately took an average of 56 days to complete.

Another common issue was interview and/or survey success rates. While several studies reported success rates as high as 60%–76% (Davis & Taylor, 1997; Davis et al., 2001; Greenspan et al., 2005; Messing et al., 2015; Pate et al., 1992), others encountered difficulty keeping respondents in the study. For instance, Stover et al. (2010) maintained only a 25% interview retention rate, while Davis et al. (2010) and Taylor (n.d.) maintained 42% and 44% retention rates, respectively. Most of this attrition was, once again, the result of an inability to establish contact with victims. To address this, Davis et al. (2010) supplemented their telephone contacts with home visits, which raised their interview success rate from 19% to 42%.

Several important but less common issues were also reported. Greenspan et al. (2005) noted problems related to the interaction between police and second responders. That is, second responders were often underutilized as police failed to summon them to domestic incidents as frequently as intended. Likewise, police officers displayed reluctance to leave second responders unattended at domestic incidents. Consequently, officers regularly remained on scene until the response concluded, which inhibited any potential time‐saving benefits of the intervention. As Hovell et al. (2006) note, the interactions between second responders and victims of family abuse are also not standardized, and it is likely that the content and nature of these interactions were variable. Accordingly, it is difficult to know if the delivery of the interventions, both within and across studies, was consistent. Finally, as is always the case, studies are likely to vary in their level of reporting validity (Perry et al., 2010). Our discussion here is inevitably limited to the issues that study authors report.

### 5.2 Risk of bias in included studies

#### 5.2.1 Study and outcome characteristics

Six items were used to assess the potential for bias in each study (Table 4). These items relate to both study and outcome characteristics and were adapted from the Cochrane randomized and non‐randomized risk of bias tools (Sterne et al., 2016; Sterne et al., 2019).^12^ At the study‐level we coded elements related to: (a) Whether random allocation was used, (b) whether significant baseline differences existed between treatment and control groups, (c) whether any implementation issues were likely to affect the results of the study, and (d) whether the analysis method was appropriate given the characteristics of the study. Items related to the outcome included: (e) Whether analysis of experimental misassignments could have impacted the outcome, and (f) whether missing outcome data might depend on its true value. Randomization and the appropriateness of the analysis were both yes or no questions, while the remaining items were coded as either “No,” “Probably no,” “Probably yes,” “Yes,” or “No information.” Studies that used appropriate analysis methods and were coded as either “No” or “Probably no” on all other items were classified as having low risk of bias. Studies that used appropriate analysis methods but were coded as either “Probably yes” or “No information” on any items were classified as having some concerns. Finally, studies that either did not use an appropriate analysis or were coded as “Yes” on any other items (aside from randomization) were classified as having high risk of bias. We note two important clarifications here. First, our risk of bias classifications involved an inherent element of subjectivity, as study authors did not directly report or determine these ratings themselves. Given that several eligible studies were conducted by authors of this review, all risk of bias determinations were made by Kevin Petersen along with a graduate research assistant at George Mason University. Second, our classifications relate only to our outcomes of interest, and do not necessarily represent the level of bias seen across all components of a study. This is an important distinction because, at times, measures of repeat family abuse were only secondary or tertiary components of a given study. For instance, Regoeczi and Hubbard (2018) focused primarily on downstream outcomes related to the prosecution and courts, only reporting on recidivism in a more limited capacity. Secondary outcomes such as these may receive less attention, and as a result, have a greater potential for bias.

**Table 4:** Risk of bias in included studies

	Randomization^a^	Nonequivalence of groups^b^	Implementation failures^c^	Appropriate analysis^d^	Misassignments^e^	Data missingness^f^	Rating
Casey et al. (2007)	No	No	No information	Yes	Probably yes	No information	Some concerns
Davis and Taylor (1997)	Yes	Probably no	Probably no	Yes	No	Probably no	Low risk
Davis et al. (2001)	Yes	Probably no	Probably yes	Yes	No	Probably no	Some concerns
Davis et al. (2010)	Yes	No	Probably no	Yes	Probably no	Probably no	Low risk
Hovell et al. (2006)	No	Probably yes	No information	Yes	Probably no	Probably no	Some concerns
Greenspan et al. (2005)	No	No	Probably yes	Yes	Probably yes	Probably no	Some concerns
Hovell et al. (2006)	No	No information	No information	Yes	Probably yes	Probably no	Some concerns
Koppensteiner et al. (2019)	Yes	Probably no	Probably no	Yes	No	Probably no	Low risk
Messing et al. (2015)	No	Probably no	Probably no	Yes	No	Probably yes	Some concerns
Mizrachi (2019)	No	Probably yes	No information	Yes	Probably no	Probably no	Some concerns
Pate et al. (1992)	Yes	No	Probably no	Yes	No	Probably no	Low risk
Regoeczi and Hubbard (2018)	No	Probably yes	Probably no	No	Probably no	Probably no	High risk
Stover et al. (2009)	No	Probably yes	Probably no	Yes	No	Probably no	Some concerns
Stover et al. (2010)	No	Probably no	Probably no	Yes	Probably yes	Probably yes	Some concerns
Taylor (n.d.)	Yes	No information	No information	No information	Probably no	Probably yes	Some concerns

^a^ Was the allocation sequence random?

^b^ Did baseline differences between intervention groups suggest a problem with the assignment process?

^c^ Were there failures in implementing the intervention that could have affected the outcome?

^d^ Was an appropriate analysis used to estimate the effect of assignment to intervention and/or control for confounding domains?

^e^ Was there potential for a substantial impact (on the result) of the failure to analyze participants in the group to which they were assigned?

^f^ Could missingness in the outcome depend on its true value?

Across our six experimental studies there was little risk of bias related to nonequivalence between treatment and control groups. While several studies did report significant post‐randomization differences, these differences were often unrelated to study outcomes. Davis and Taylor (1997) noted that there were significantly more female offenders and victims abused by children assigned to the treatment group, but neither of these variables appeared to impact the prevalence of repeat abuse reported to the police. In a similar fashion, Davis et al. (2001) found that treatment victims were more likely to be Hispanic and that treatment offenders were more likely to be both Hispanic and male, however, neither factor consistently predicted repeat abuse. Lastly, Koppensteiner et al. (2019) reported that control victims were more likely to be living with their offender and that control offenders were more likely to be unemployed. While there were no direct tests of the relationship between these factors and the outcome measures, it is likely that, based on prior research, any bias resulting from these differences would have favored the control group (see Sherman, 2018). Additionally, significant differences between groups in these studies did not appear to be more prevalent than would be expected by chance. Each study reported nonsignificant differences between groups on the majority of characteristics that were tested, and thus it is only natural for a small proportion of significant differences to emerge across a large number of significance tests.

More notable baseline differences emerged across our nine quasi‐experimental studies. Two studies reported significant differences between treatment and control groups on a variety of factors potentially relevant to our outcome measures. Stover et al. (2009) noted significant differences between groups on variables such as ethnicity, relationship status, and the severity of the triggering offense. Specifically, control cases involved significantly more severe triggering offenses and significantly fewer bidirectionally violent incidents (i.e., dual violent arrests). While these differences appeared to favor the treatment group, other differences were more ambiguous or tended to favor the control group. For instance, treatment cases were more likely to involve married or cohabitating couples, Hispanic victims, and victims with limited English language proficiency. Similarly, Regoeczi and Hubbard (2018) reported that treatment offenders in their study had significantly longer criminal histories, were significantly more likely to have a protection order out against them, were significantly more likely to have used a weapon, and were significantly more likely to have inflicted visible injury on the victim. Recidivism was also a secondary outcome in their study, and the analysis did not appear to control for any of these potentially confounding factors.

Two studies also reported aspects of their allocation process that made nonequivalence likely. The interventions evaluated by Friday et al. (2006) and Mizrachi (2019) were intended only for severe or high‐risk domestic abuse cases and victims. Both studies employed a comparison group of cases that did not receive the intervention, seemingly based on the characteristics of the victim and/or incident itself. For instance, Friday et al. compared intervention cases to cases that were rejected by the intervention team. Subsequent analysis confirmed that that the intervention unit was selecting only the most severe cases. Similarly, Mizrachi employed a comparison group of cases that were deemed ineligible to receive the intervention by the responding officer, and further analyses suggested that (among other things) the provision of the intervention was significantly correlated with indicators of risk.^13^ To address these issues, both studies attempted to control for selection bias during analysis.

There was often limited information from which to determine whether implementation failures may have biased study results, however, two studies did report difficulties that likely impacted our outcomes of interest. As discussed in the prior section, Davis et al. (2001) encountered significant issues successfully locating victims, leading to a low face‐to‐face contact rate and substantial delays in the delivery of the intervention, while police officers in Greenspan et al.'s (2005) study frequently failed to summon second responders to domestic incidents. Greenspan et al. were unable to determine the criteria that officers used in selecting cases for second responses, and so it was difficult to determine the level of bias that this may have introduced.

In evaluating the severity of treatment misassignments, we were primarily focused on the degree to which treatment crossovers were excluded from the analysis. While many studies used either a strict intent‐to‐treat approach (Davis & Taylor, 1997; Davis et al. 2001; Koppenstiener et al., 2019; Pate et al., 1992) or excluded only a small number of misassignments (Davis et al., 2010), others appeared to analyze only cases where the full treatment was delivered. This was particularly true for studies that involved post‐hoc comparisons of cases that received a second response to those that did not (Casey et al., 2007; Hovell et al., 2006), and for studies that were limited to victim‐reported measures of repeat abuse only (Greenspan et al., 2005; Stover et al., 2010). In these situations, it remains possible for the exclusion of cases where treatment was attempted but not delivered to impact study outcomes. Victims and/or households who are not successfully contacted by second responders may differ in their propensity to experience repeat victimization, and the exclusion of these households from any subsequent analyses may lead to bias. However, we also note that it was difficult at times to determine the degree to which otherwise eligible cases may have been excluded from a study due to issues with treatment delivery, and our determinations here are based on our interpretation of the details reported in each study.

A final concern in our eligible studies was the potential for outcome measures to be correlated with nonresponse bias. That is, victims who were lost to follow‐up may have differed in their propensity to experience repeat victimization. Several studies experienced low survey success rates but were able to demonstrate similarity between victims who were interviewed and those who were not, or alternatively, noted similar nonresponse rates between treatment and control groups (see Davis et al., 2010; Koppensteiner et al., 2019). However, two studies reported significant differences between surveyed victims and non‐surveyed victims, specifically on variables such as employment, education, charge severity, and relationship status (Messing et al., 2015; Stover et al., 2010). One additional study reported a low survey success rate but did not report any information to suggest similarity between the victims who were surveyed and those who were not (Taylor, n.d.).

### 5.3 Synthesis of results

#### 5.3.1 Synthesis of results

Fifteen studies were analyzed across two main outcome constructs and one secondary outcome construct, resulting in the calculation of 29 effect sizes (including sensitivity analyses). For dichotomous outcomes, results were converted to percentage point differences by subtracting 1 from the odds ratio and multiplying by 100. Thus, odds ratios greater than 1 indicate an increase in the odds of a given outcome for treatment groups relative to control groups.

The combined mean effect sizes for each outcome are displayed in Table 5. We also chose to display separate estimates for experiments and quasi‐experiments as a component of our main results given the importance of this distinction to the findings of the prior review (Davis et al., 2008). In our combined models, only the secondary outcome of victim use‐of‐services reached statistical significance. Specifically, these results suggest that second responder programs increase the likelihood that victims will access and use support services after a family abuse incident, though only a small number of studies were included in this analysis.^14^ Results for both official and unofficial measures of repeat family abuse were not statistically significant and displayed mean effect size estimates that were largely null (i.e., no difference between groups). When estimating separate models for experiments and quasi‐experiments, however, the results are more suggestive of a treatment effect. That is, the results of our six experimental studies suggest that second responder interventions may significantly increase the odds that a repeat incident of family abuse is reported to the police, though these studies are not suggestive of an equally significant increase in victim‐reported incidents. Taken together, our findings suggest both intended and (possibly) unintended effects of second responder programs, yet these findings are also sensitive to the rigor of the research being conducted. Below we discuss our results for each outcome measure in more detail, along with visual depictions of our treatment estimates.

**Table 5:** Summary effects for all studies, experiments, and quasi‐experiments

Outcome	OR (% difference)	95% CI	*Q*	*I* ^2^	*τ* ^2^	*k*	*Q*_model_ (*p*‐value)
Repeat family abuse (police data)							
Combined	1.03 (3%)	0.82, 1.30	35.19***	75.49	0.11	12	2.06
Experimental	1.22* (22%)	1.04, 1.43	2.84	0.00	0.00	6	(*p* = 0.15)
Quasi‐experimental	0.86 (−14%)	0.55, 1.35	28.39***	86.98	0.25	6	
Repeat family abuse (victim reported)							
Combined	1.00 (0%)	0.80, 1.24	10.14	23.74	0.02	8	3.29
Experimental	1.15 (15%)	0.88, 1.50	4.03	12.59	0.01	5	(*p* = 0.07)
Quasi‐experimental	0.81 (−19%)	0.62, 1.06	2.73	0.00	0.00	3	
Victim use of services							
Combined	1.09* (9%)	1.02, 1.16	1.19	0.00	0.00	4	0.45
Experimental	1.21 (21%)	0.88, 1.64	0.65	0.00	0.00	2	(*p* = 0.50)
Quasi‐experimental	1.08* (8%)	1.01, 1.16	0.08	0.00	0.00	2	

*Note*: The *Q_model_* statistics in this table use separate estimates of *τ* ^2^ for each group.

Abbreviations: CI, confidence interval; *Q*, test for heterogeneity; *I* ^2^, percentage of variability due to between‐study heterogeneity; *τ* ^2^, random effects variance component; *k*, number of effect sizes; *Q*_model_, test for whether significant heterogeneity is explained by research design (experimental vs. quasi‐experimental); OR, odds ratio.

* *p* < 0.05

*** *p* < 0.001.

#### 5.3.2 Repeat family abuse based on police data

While there has been debate regarding how to interpret increased victim contact with police following second responder interventions (i.e., favors treatment or control), the long‐term goal of these programs is to reduce measures of repeat family abuse (Davis & Taylor, 1997; Hovell et al., 2006). As such, we consider increases in the odds of repeat abuse based on official data sources as effects favorable to control. However, it is important to acknowledge that family abuse is significantly underreported (Felson & Paré, 2005; Strong & Cohen, 2013), and thus increased reporting could be considered a beneficial outcome, particularly in the short‐term.

**Figure 2:**
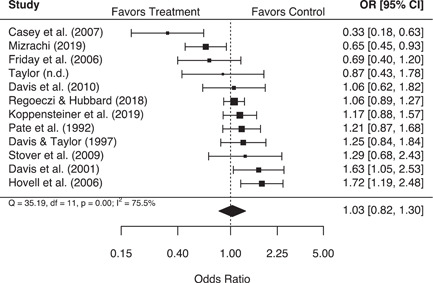
Effect of second responder interventions on repeat family abuse (police data). CI, confidence interval; OR, odds ratio

Twelve of our 15 studies reported a measure of repeat family abuse based on official data sources. The forest plot for these studies can be seen in Figure 2, which displays the effect size for each study, the confidence intervals for the effect size, the no effect or reference line, and the mean effect size across all studies included in the model. Given that our desired effect here is a reduction in officially reported recidivism, estimates falling to the left of the reference line are considered favorable to treatment, while estimates falling to the right of the line are considered favorable to control. The size of the square points for each study represents the amount of weight that the study is given in the overall analysis, which is inversely related to the variance of the effect size estimate (Lipsey & Wilson, 2001). The mean effect size for official measures of recidivism is 1.03, indicating a 3% increase in the odds of a police‐reported repeat family abuse incident for treatment groups relative to control groups. While this effect favors control groups, it is not statistically significant, with confidence intervals ranging from an 18% decrease to a 30% increase in the odds of a repeat incident. There is also a significant amount of heterogeneity in this model, with roughly three quarters of the total variability being attributable to between‐study variance.

**Figure 3:**
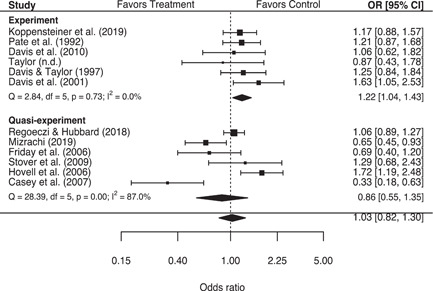
Effect of second responder interventions on repeat family abuse by research design (police data). CI, confidence interval; OR, odds ratio

As suggested by prior research (Shadish & Heinsman, 1997; Weisburd et al., 2001), along with the findings of the original review on this topic (Davis et al., 2008), effect sizes may vary considerably by methodological rigor. Figure 3 separates the effects of second responder interventions on official measures of recidivism by research design. When estimated separately, findings from experimental studies suggest a statistically significant 22% increase in the odds of a police‐reported repeat family abuse incident for treatment groups relative to control groups. In contrast, quasi‐experimental studies were associated with a nonsignificant 14% decrease in the odds of a repeat incident. While notably discrepant, the mean effect sizes for experimental and quasi‐experimental studies did not significantly differ, as indicated by the nonsignificant *Q_model_* statistic reported in Table 5. There are also notable differences in the amount of heterogeneity associated with experimental and quasi‐experimental studies. While 87% of the variance in quasi‐experimental results was attributable to between‐study heterogeneity, all observed variability in experimental studies could be attributed to sampling error alone. Accordingly, the experimental model does not contain a random effects variance component, and thus the distribution resembles that of a fixed or common effect.

#### 5.3.3 Repeat family abuse based on victim reports

**Figure 4:**
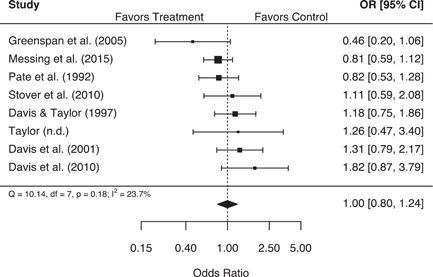
Effect of second responder interventions on repeat family abuse (victim data). CI, confidence interval; OR, odds ratio

Victim‐reported repeat abuse is often considered to be a more accurate measure of true prevalence rates than official data sources, addressing the high levels of under‐reporting seen with official data sources. Thus, victimization reports may be a particularly important indicator of the efficacy of second responder interventions (Greenspan et al., 2005; see also Casey et al., 2007; Hovell et al., 2006). Eight of our eligible studies reported a measure of repeat family abuse taken from victim interviews or surveys, and the forest plot for these studies can be seen in Figure 4. The overall mean effect size is null, indicating that that the odds of a victim‐reported repeat family abuse incident were approximately equal for treatment and control groups.^15^ Consequently, this effect is not statistically significant, and the confidence intervals range from a 20% decrease to a 24% increase in the odds of a repeat incident. There is a lack of significant heterogeneity for these studies as well, and less than a quarter of the total variability is attributable to between‐study variance. As reported in Table 5, notable differences did exist between experimental and quasi‐experimental studies for this outcome. While quasi‐experimental studies were associated with a 19% decrease in the odds of a victim‐reported repeat family abuse incident, experimental studies were associated with a 15% increase. However, these estimates were neither significantly different from 1 nor from either other, and we do not display separate plots for these models as we did with our police‐reported measures.

#### 5.3.4 Use of victim services

One of the primary mechanisms through which second responder interventions may reduce future victimization is through the provision of victim services (Davis et al., 2008; Mazerolle et al., 2018). Accordingly, for these programs to evidence effectiveness we may expect to see increases in service use among victims who received the response, compared to those who did not. It worth reiterating here that service use was included only as a secondary outcome in this review. All studies were still required to report a measure of repeat abuse and we did not conduct dedicated searches or screenings for studies that measured service use only. Nonetheless, we calculated effect sizes for four eligible studies that compared the use of victim services between treatment and control groups following the intervention.

**Figure 5:**
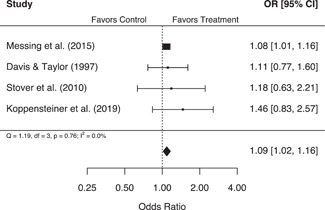
Effect of second responder interventions of use of victim services. CI, confidence interval; OR, odds ratio

The forest plot for this outcome is shown in Figure 5. In contrast to measures of repeat abuse, here we interpret increases in the outcome as intended effects, and thus effect sizes positioned to the right of the reference line are considered effects favorable to treatment. The mean effect size of 1.09 represents a 9% increase in the odds of service use for treatment victims relative to control victims following the second responder intervention. There is a lack of significant or excess heterogeneity in these effect sizes, and all studies report results that favor the treatment group. When separating experimental and quasi‐experimental studies (Table 5), quasi‐experimental studies are associated with a statistically significant 8% increase in the odds of service use, and experimental studies are associated with a nonsignificant 21% increase. Experimental studies in this model (Davis & Taylor, 1997; Koppensteiner et al., 2019) display notably more within‐study variance and receive less weight in the combined analysis. However, there are a small number of studies in each group, and both sets of studies display results favoring a treatment effect. Accordingly, we would urge caution interpreting any differential effects for this outcome by research design, and present these results in Table 5 only to remain consistent with our other outcome measures.

#### 5.3.5 Sensitivity analysis results

As previously noted, we ran several sensitivity analyses across our three outcome measures. First, we estimated the effect of second responder interventions on police‐reported repeat abuse substituting the shortest follow‐up duration for the longest follow‐up duration for two studies that allowed us to calculate multiple effect sizes (Hovell et al., 2006; Koppenstiener et al., 2019). The overall results for this model were substantively similar to those using the longest follow‐up period, with an overall mean effect size of 0.98, suggesting a nonsignificant 2% decrease in the odds of a police‐reported repeat family abuse incident compared to a nonsignificant 3% increase in the original analysis. Results for quasi‐experimental studies were also similar, changing from a nonsignificant 14% decrease in the odds of a repeat incident in the original analysis to a nonsignificant 19% decrease in the odds of a repeat incident in the sensitivity analysis. However, results for experimental studies using these effect sizes suggested only a marginally significant 15% (*p* = 0.08) increase in the odds of a police‐reported repeat incident, compared to a statistically significant 22% increase in the original analysis. While the story is qualitatively similar, with experimental studies suggesting notable increases in officially reported repeat victimization and quasi‐experimental studies suggesting notable decreases, the significance of the findings from experimental studies do seem to be sensitive to the choice of these effect sizes. We explore the impact of follow‐up duration as a moderator variable in the following section.

Two studies reporting on official measures of repeat abuse also allowed us to calculate effect sizes for the entire treatment group as well as for those who engaged with specific aspects of the treatment (Friday et al., 2006; Stover et al., 2009). We prioritized the general treatment effects in our primary model, but we re‐ran this model using the more specific treatment estimates, such as estimates for those who successfully spoke with second responders or met with domestic violence counselors. Again, overall results were similar, suggesting a statistically nonsignificant 7% increase in the odds of a police‐reported repeat family abuse incident for treatment groups relative to control groups, compared to a nonsignificant 3% increase in the original analysis. Using these effect sizes, experimental studies continued to be associated with a significant 22% increase in the odds of a police‐reported repeat incident, and quasi‐experiments were associated with a nonsignificant 9% decrease in the odds of a repeat incident.

Finally, one study allowed us to calculate a separate effect size for victim use‐of‐services limited to only those victims who received treatment (Messing et al., 2015). We re‐ran the victim services model using this effect size, and the results of this model suggested a statistically significant 29% (*p* = 0.03) increase in the use of victim services for treatment groups compared to control groups, compared to only a 9% increase in the original analysis. Unsurprisingly then, restricting this effect size to only those victims who were successfully treated led to a notable increase in the mean effect size, though in both models the effect was statistically significant and favored treatment. We also explore the potential for successful treatment delivery (as measured by face‐to‐face contact rate) to moderate effect sizes across studies in the following section.

### 5.4 Additional moderator analyses

Second responder interventions face a number of obstacles that may impact the effects that they produce. Among others, these interventions seek to capitalize on a short period of opportunity after an incident of family abuse occurs, but often encounter delays in the delivery of the response (Davis & Medina, 2001). They seek to reduce future victimization in the long‐term, but with the understanding that short‐term effects may include increased victim willingness to call police (Davis & Taylor, 1997). Second responders must also successfully locate and contact victims to deliver the intervention. However, many victims may refuse the response or prove difficult to locate (Davis et al., 2001; Davis et al., 2010; Stover et al., 2010). With these considerations in mind, we conducted several additional post‐hoc moderator analyses beyond the separate analysis of experimental and quasi‐experimental studies reported above. Specifically, we examined the effect of the amount of time that elapses between the incident and the second response, the length of the follow‐up period, the type of family abuse complaints and police responses included, the face‐to‐face contact and survey success rates, the unit of analysis used in the study, and the presence of confounding interventions. We conducted these moderators only on our more common police and victim‐reported measures of repeat family abuse.^16^ Even still, the sample sizes for many of these analyses are small and should be interpreted with caution. All categorical moderators were conducted using the analog to the ANOVA method on the combined models presented earlier, while continuous moderators were analyzed using meta‐regression (Higgins et al., 2020; Lipsey & Wilson, 2001).

#### 5.4.1 Speed of the second response

The more immediately that a second response can occur after an incident of family abuse, the more optimal the results may be (Davis et al., 2010; Greenspan et al., 2005; Messing et al., 2015). Table 6 shows the effect of response speed on repeat family abuse for both police and victim‐reported data. While we coded this variable as either occurring within 72 h, within 1 week, or more than one week after the triggering incident, we treated this as a linear relationship and our results are reported using regression coefficients. For official data measures, there was no significant linear effect of the speed of the second response, with the odds of a police‐reported repeat incident increasing only slightly the longer the response took. However, for victim reported measures, there was a significant relationship between the speed of the response and the odds of a repeat incident of family abuse. Specifically, increases in the amount of time that elapsed between the triggering incident and the second response were associated with increased odds of reporting an incident. When the second response occurred with 72 h, there was a predicted 19% decrease in the odds of a victim‐reported repeat incident for treatment groups relative to control groups. In contrast, when the second response occurred more than a week after the incident, there was a predicted 24% increase in the odds of a victim‐reported repeat incident for treatment groups relative to control groups.

**Table 6:** Effect of second responder interventions by speed of response

Outcome	Level	*k*	OR (% difference)	95% CI	Regression coefficient	*z* (*p‐*value)
Police data	72 h	5	1.04 (4%)	0.73, 1.46	0.027	0.17
	1 week	3	1.07 (7%)	0.82, 1.39		(*p* = 0.87)
	More than 1 week	3	1.09 (9%)	0.69, 1.73		
Victim data	72 h	3	0.81 (−19%)	0.63, 1.05	0.212	2.00*
	1 week	2	1.01 (1%)	0.84, 1.21		(*p *= 0.045)
	More than 1 week	3	1.24 (24%)	0.92, 1.67		

Abbreviations: CI, confidence interval; OR, odds ratio.

* *p *< 0.05.

It is important to note that these results represent a linear relationship and not a direct comparison of any two categories of response timing. As the delivery of these interventions becomes slower, the effect sizes shift from favoring treatment to favoring control. This does not indicate that the mean effect sizes between any two levels are significantly different from each other, and indeed the confidence intervals for each level overlap (for a similar discussion of this issue see Lum et al., 2020). Nonetheless, these findings support the notion that second responder interventions may need to capitalize on a small window of opportunity to produce their desired effects, and that these effects may be seen in victim‐reported measures rather than official data measures.

#### 5.4.2 Length of follow‐up period

Second responder interventions may serve to increase victim confidence and satisfaction with police, leading to increases in victim willingness to call the police when an incident occurs (Greenspan et al., 2005; Koppensteiner et al., 2019; Stover et al., 2010). However, over time the goal is that these programs will lead to a reduction in repeat calls to police (Davis & Taylor, 1997), and thus the length of the follow‐up period used to evaluate second responder programs may impact study results. Table 7 displays the linear relationship between the duration of follow‐up (measured in months) and both police and victim‐reported repeat family abuse. While each one month increase in follow‐up duration is associated with a 1.3% decrease in the odds of a police‐reported repeat family abuse incident and a 3.7% decrease in the odds of a victim‐reported repeat incident, neither effect approaches statistical significance.

**Table 7:** Effect of second responder interventions by follow‐up duration

Outcome	*k*	Regression coefficient	95% CI	*z* (*p*‐value)
Police data	12	−0.013	−0.03, 0.01	−1.21 (*p* = 0.23)
Victim data	8	−0.038	−0.18, 0.10	−0.55 (*p* = 0.59)

Abbreviation: CI, confidence interval.

#### 5.4.3 Type of complaint

It has been suggested that variation in the type of family abuse complaints included in second responder evaluations may mask important outcome differences (see Davis & Maxwell, 2002; Davis et al., 2006). For example, elder abuse and general family abuse incidents may be associated with unique issues related to financial and physical dependence, making them more resistant to intervention (Davis & Medina, 2001). In Table 8, we compare the mean effect sizes for studies that included only intimate partner violence incidents with those that included more general measures of family or elder abuse. For police‐reported repeat abuse, there were no significant differences between these groups. However, for victim‐reported measures, the difference between groups was marginally significant. Studies that included only intimate partner violence cases experienced a 13% decrease in the odds of a victim‐reported repeat incident, while studies that included more general measures of family abuse were associated with a 24% increase.

**Table 8:** Effect of second responder interventions by type of complaint

Outcome	Level	*k*	OR (% difference)	95% CI	*Q*_model_ (*p*‐value)
Police data	Elder/general family	5	1.11 (11%)	0.76, 1.62	0.27 (*p* = 0.60)
	Intimate partner only	7	0.97 (−3%)	0.71, 1.33	
Victim data	Elder/general family	3	1.24 (24%)	0.90, 1.71	3.11+ (*p* = 0.08)
	Intimate partner only	5	0.87 (−13%)	0.70, 1.09	

*Note*: *Q*_model_ tests whether a significant amount of heterogeneity is explained by the moderator.

Abbreviations: CI, confidence interval; OR, odds ratio.

^+^ *p* < 0.10.

#### 5.4.4 Type of police response

Whether an offender is arrested following a family abuse incident has been shown to exert important independent effects on recidivism (Sherman, 2018; Sherman et al., 1992).^17^ Thus, it remains possible that the impact of second responder interventions on repeat abuse is moderated by the way that the police respond to the initial complaint. Given this potential, in Table 9 we compare the effect sizes for studies in which the second response was limited to arrests with those that included other types of family abuse reports, such as founded or unfounded reports. Results indicated that there was a notable observed decrease in the odds of a future police‐reported incident for studies using arrests only, yet this was coupled with a notable increase in the odds of a future victim‐reported incident for these studies. Neither difference was statistically significant, however.

**Table 9:** Effect of second responder interventions by police response

Outcome	Level	*k*	OR (% difference)	95% CI	*Q*_model_ (*p*‐value)
Police data	Any report	9	1.12 (12%)	0.88, 1.42	2.41 (*p* = 0.12)
	Arrests only	3	0.71 (−29%)	0.43, 1.19	
Victim data	Any report	6	0.98 (−2%)	0.76, 1.25	0.27 (*p* = 0.60)
	Arrests only	2	1.16 (16%)	0.64, 2.09	

*Note*: *Q*_model_ tests whether a significant amount of heterogeneity is explained by the moderator.

Abbreviations: CI, confidence interval; OR, odds ratio.

#### 5.4.5 Face‐to‐face contact rate

Second responder interventions often suffer from difficulty locating and contacting victims (Davis et al., 2001; Stover et al., 2009), and it is possible that victims who cannot be successfully contacted have a greater propensity for revictimization. Table 10 tests the linear relationship between face‐to‐face contact rate and repeat family abuse for the studies where a contact rate could be discerned. Of note, we only examine this moderator variable for police‐reported repeat family abuse incidents, as victim‐reported measures were inevitably derived from victims who received treatment and were successfully contacted. Our findings suggest that there was no significant relationship between face‐to‐face contact rate and police‐reported repeat family abuse.^18^

**Table 10:** Effect of second responder interventions by face‐to‐face contact rate

Outcome	*k*	Regression coefficient	95% CI	*z* (*p*‐value)
Police data	8	−0.009	−0.019, 0.002	−1.63 (*p* = 0.10)

Abbreviation: CI, confidence interval.

#### 5.4.6 Survey success rate

It is also possible that victim attrition from follow‐up surveys impacts study results. Those who remain in the study may be more likely to access and utilize services, leave their abusers, or take other measures to reduce their propensity for future victimization. We examine the relationship between survey success rate and victim‐reported repeat victimization in Table 11. While results suggested that increases in survey success rates were associated with decreases in the odds of victim‐reported repeat abuse, this relationship was not statistically significant.

**Table 11:** Effect of second responder interventions by survey success rate

Outcome	*k*	Regression coefficient	95% CI	*z* (*p*‐value)
Victim data	8	−0.008	−0.02, 0.01	−0.99 (*p* = 0.32)

Abbreviation: CI, confidence interval.

#### 5.4.7 Unit of analysis

Studies limiting their units of analysis to the same victim/offender pairing or household may report different findings than those that follow the same victim or offender more generally. A central objective of second responder programs is to provide victims with the tools to leave their abusers, should they choose to do so (Davis et al., 2010; Koppensteiner et al., 2019). If victims do successfully leave, they inevitably reduce their chances of reporting victimization involving the same offender. However, research has consistently shown that repeat victimization follows a nonrandom distribution, often predicted by prior abuse and lifestyle characteristics (see Dowling & Morgan, 2019; Turanovic & Pratt, 2014). Thus, victims who leave their abusers may enter into new abusive relationships during the study follow‐up period, and this abuse could be lost when recording only those incidents that occur within the same household or involving the same couple. Table 12 compares the mean effect sizes between these two groups of studies. The results indicate relatively large and statistically significant differences in the mean effect sizes for police‐reported repeat abuse. While studies that followed the same household or victim/offender pairing experienced a statistically significant 28% increase in the odds of a repeat incident, studies that followed the same victim or offender more generally experienced a 22% decrease in the odds of a repeat incident, compared to control groups. Similar findings are observed for victim‐reported repeat abuse, with studies following the same household or victim/offender pairing experiencing a 14% increase in the odds of a repeat incident and studies following the same victim or offender more generally experiencing a 19% decrease in the odds of a repeat incident, though these results are only marginally significant.

**Table 12:** Effect of second responder interventions by general unit of analysis

Outcome	Level	*K*	OR (% difference)	95% CI	*Q*_model_ (*p*‐value)
Police data	Any pairing	5	0.78 (−22%)	0.59, 1.03	7.31** (*p* = 0.01)
	Same pairing/household	7	1.28 (28%)	1.02, 1.61	
Victim data	Any pairing	3	0.81 (−19%)	0.61, 1.08	3.11+ (*p* = 0.08)
	Same pairing/household	5	1.14 (14%)	0.89, 1.48	

*Note*: *Q*_model_ tests whether a significant amount of heterogeneity is explained by the moderator.

Abbreviations: CI, confidence interval; OR, odds ratio.

^+^ *p* < 0.10;

** *p* < 0.01.

These findings may seem surprising, as measures of repeat abuse involving any offender should be the more comprehensive outcome measure and should be more sensitive to the potential for victims to enter into new abusive relationships during the study period. An alternative explanation, however, is that family abuse is increasingly persistent at the household level. Victims who successfully leave their abusers might report a reduction in repeat victimization, but the offender associated with the household may continue to abuse. This new abuse could simply displace to other family members or new intimate partners, and consequently still be attributed to the same household. Alternatively, studies measuring repeat abuse at the household level may be increasingly likely to capture incidents committed or reported by other household members. There is strong evidence to indicate a significant victim/offender overlap in abusive domestic relationships (Iratzoqui, 2018; Muftić et al., 2015; Tillyer & Wright, 2014). If second responder interventions increase willingness to report family abuse, these effects may diffuse throughout the entire household, leading to increased reporting of incidents committed and/or reported by other family members. If so, we might expect to see studies that analyze repeat abuse at the household‐level to report less favorable results than those that analyze individual victims or victim/offender pairings.

We explore this potential in Table 13, though we only do so for police‐reported repeat abuse, given that survey measures in these studies were all taken from the same victim as the original offense. For police‐reported repeat abuse, studies that analyzed households were associated with significantly higher odds of a repeat incident than studies that analyzed individuals or couples. Household studies reported a 39% increase in the odds of a police‐reported repeat incident, while studies analyzing individuals or couples reported a 9% decrease in the odds of a repeat incident, relative to control groups. Taken together, these results suggest the importance of the unit of analysis used in second responder evaluations. It is possible that measuring repeat abuse at the household‐level may be less sensitive to the potential for victims to relocate and leave their abusive environments, or that second responder interventions have increased reporting effects for multiple household members. However, it should be made clear that these interpretations are merely speculative. We are not able to consistently discern whether victims left their abusers following the second responder intervention and how this may have impacted study outcomes. Additionally, we are unable to determine who was responsible for making the initial or repeat police reports in our eligible studies.^19^ These limitations prevent any strong inferences with regard to the interpretation of this moderator analysis, and it remains ambiguous as to what led to these findings.

**Table 13:** Effect of second responder interventions by households versus victims/couples

Outcome	Level	*K*	OR (% difference)	95% CI	*Q*_model_ (*p*‐value)
Police data	Victim/couple	8	0.91 (−9%)	0.71, 1.15	3.86* (*p* = 0.049)
	Household/family	4	1.39 (39%)	0.98, 1.98	

*Note*: *Q*_model_ tests whether a significant amount of heterogeneity is explained by the moderator.

Abbreviations: CI, confidence interval; OR, odds ratio.

* *p *< 0.05.

#### 5.4.8 Confounding interventions

Finally, several of our eligible studies included components other than the second response that could be considered confounding. These components included the use of validated risk assessment screenings (Messing et al., 2015; Mizrachi, 2019), increased investigation and/or prosecution (Friday et al., 2006; Regoeczi & Hubbard, 2018), and public education campaigns (Davis & Taylor, 1997; Davis et al., 2001). Given the potential for confounding in these studies, we compared their mean effect sizes to those of studies that involved only a second responder intervention. As seen in Table 14, separating study effects in this way did not indicate any significant differences for either police or victim‐reported measures of repeat family abuse.

**Table 14:** Effect of confounding interventions on study outcomes (general)

Outcome	Level	*k*	OR (% difference)	95% CI	*Q*_model_ (*p*‐value)
Police data	Second response only	7	1.05 (5%)	0.75, 1.45	0.03 (*p* = 0.86)
	Additional interventions	5	1.00 (0%)	0.70, 1.44	
Victim data	Second response only	5	0.98 (−2%)	0.68, 1.39	0.06 (*p* = 0.81)
	Additional interventions	3	1.04 (4%)	0.74, 1.46	

*Note*: *Q*_model_ tests whether a significant amount of heterogeneity is explained by the moderator.

Abbreviations: CI, confidence interval; OR, odds ratio.

However, given that the type of confounding interventions across these studies varied, we also conducted a moderator analysis separating confounding interventions into more homogenous piles (Table 15). Results suggest that, for both police and victim‐reported repeat abuse, second responder programs that included a public education campaign produced the largest increases in the odds of a repeat incident, while programs including a lethality assessment screening produced the largest decreases. However, there were no significant differences between categories and no significant effects for any single category. Additionally, this test included very small sample sizes, and the interventions that incorporated a public education campaign both involved the same program and occurred in the same location (Davis & Taylor, 1997; Davis et al., 2001).

**Table 15:** Effect of confounding intervention on study outcomes (specific)

Outcome	Level	*K*	OR (% difference)	95% CI	*Q*_model_ (*p*‐value)
	Second response only	7	1.05 (5%)	0.77, 1.44	2.93 (*p* = 0.40
Police data	Lethality assessment	1	0.65 (−35%)	0.30, 1.39	
	Public education	2	1.42 (42%)	0.81, 2.48	
	Investigation/prosecution	2	0.90 (−10%)	0.52, 1.54	
Victim data	Second response only	5	0.96 (−4%)	0.72, 1.29	1.84 (*p* = 0.24)
	Lethality assessment	1	0.81 (−19%)	0.57, 1.15	
	Public education	2	1.24 (24%)	0.87, 1.76	
	Investigation/prosecution	0			

*Note*: *Q*_model_ tests whether a significant amount of heterogeneity is explained by the moderator.

Abbreviations: CI, confidence interval; OR, odds ratio.

### 5.5 Publication bias

The “file‐drawer” (Rosenthal, 1979, p. 638) problem, or the potential for selective publication of studies based on their findings is a major concern associated with systematic reviews (Rothstein, 2008). We attempted to limit this potential through our comprehensive search strategies, which yielded seven (46.7%) studies classified as either government reports, dissertations, or other forms of unpublished manuscripts. Nonetheless, there is always potential to miss unpublished studies, and for these omissions to bias the results of the analyses. Our assessment of publication bias is focused on our combined models of police and victim‐reported incidents of repeat family abuse, given the scarcity of studies that measured the use of victim services.

**Figure 6:**
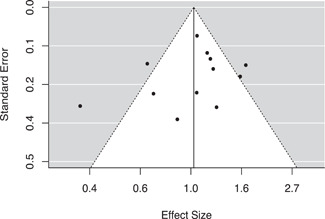
Funnel plot: Police‐reported repeat abuse

Simple comparison of mean effect sizes for published journal articles versus government reports, dissertations, or other unpublished manuscripts suggests that published articles were associated with slightly higher odds of a police‐reported repeat family abuse incident (odds ratio [OR] for published articles = 1.05; OR for unpublished articles = 1.00), however, this difference is not statistically significant (*Q*_model_ = 0.04, *p* = 0.84). The funnel plot for this outcome can be seen in Figure 6. While there appears to be a slight asymmetry toward the right side of the plot as the standard error increases, a trim‐and‐fill analysis did not detect any significant asymmetry and did not impute any additional effect sizes. Furthermore, an Egger's test for the linear relationship between effect size and standard error was nonsignificant (*t* = −0.72, *p* = 0.49).

**Figure 7:**
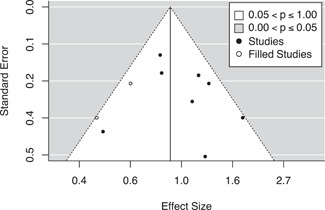
Funnel plot: Victim‐reported repeat abuse

For victim‐reported measures of repeat family abuse, the results are similar. There was a slightly higher mean effect size for published articles (OR = 1.08) than for unpublished reports (OR = 0.92), but this difference was not statistically significant (*Q*_model_ = 0.41, *p* = 0.52). The funnel plot for this outcome (Figure 7) shows an asymmetry toward the left side of the plot. This was confirmed by the trim‐and‐fill analysis, which imputed two effect sizes to the left of the reference line. These imputed studies shifted the mean effect size for this model from approximately null (OR = 1.00) to a 10% decrease in the odds of a victim‐reported repeat incident (OR = 0.90) for treatment groups relative to control groups, though this effect remained nonsignificant (*z* = −0.90, *p* = 0.37). Additionally, an Egger's regression test did not suggest any significant asymmetry (*t* = 0.73, *p* = 0.50).

In sum, the results of our moderator analyses, trim‐and‐fill analyses, and Egger's regression tests indicate that any potential publication bias in our review was likely minor. The only indication of potential bias occurred for our victim‐reported measures of repeat abuse, where the imputed studies did not significantly alter our mean effect size. Thus, it does not appear as though publication bias meaningfully altered the findings of this review.

## 6 DISCUSSION

### 6.1 Summary of main results

Analyzing 29 effect sizes across 15 studies, the results of this updated review remain largely consistent with those of the prior one. While across all studies we do not find a significant impact on reporting, in experimental studies, second responder interventions were associated with a statistically significant 22% increase in the odds of a police‐reported repeat family abuse incident. Additionally, we failed to detect any significant impact of second responder interventions on the odds of a victim‐reported repeat family abuse incident, at least in the aggregate. We do, however, find evidence to suggest that second responder interventions may significantly increase the likelihood of victims accessing domestic abuse services after an incident. This would be considered an intended effect of the intervention, and perhaps an effect that would be necessary to produce eventual impacts on the prevalence of repeat family abuse. Despite this, it is not clear based on our findings that having access to or receiving these services is sufficient to lead to long‐term reductions in victimization.

Importantly, our results point to several characteristics of second responder programs that may significantly moderate their observed impact on repeat family abuse. Increases in the speed of the second response (i.e., time elapsed between incidents and intervention) were associated with significant decreases in the odds of a victim‐reported repeat incident, suggesting the importance of the short “window of opportunity” that these interventions seek to capitalize on. This effect was limited to victim‐reported measures, however, and to the degree that increased reporting of repeat family abuse incidents is an intended outcome, the speed of the second response did not appear to be a significant determinant of treatment effects. A potential explanation for this discrepancy is suggested by Weisburd et al. (2021) in a recent study on community policing and crime prevention, where there was an apparent inflation in crime reporting by citizens that offset the ability of official data to accurately reflect the crime prevention effects of the program. A similar mechanism may be involved here, where the effect of the treatment observed in the self‐report data masks any observed effect in the police‐report data. In other words, the speed of the second response may increase police‐reported repeat abuse, but this effect could be difficult to detect given a reduction in the true prevalence of repeat abuse.

We also find some evidence to suggest that the unit of analysis chosen by second responder studies impacts the observed odds of a police‐reported repeat family abuse incident. Studies that limit their outcomes to incidents occurring within the same household report higher odds of a repeat incident than studies that follow the same victim or victim/offender pairing more generally. It remains possible that limiting outcome measures to abuse occurring within the same household may lead to the exclusion of victims who successfully leave these environments following the intervention, or that an increased sensitivity and willingness to report family abuse incidents occurs across multiple family/household members. While plausible, this is merely speculation, and is unclear why the unit of analysis appears to impact study results.

We also failed to find significant effects of several other moderating characteristics, such as follow‐up duration, face‐to‐face contact and survey success rates, and the type of complaints or police responses included. While not significant, several of these characteristics trended toward significance, or displayed divergent effect size estimates. Specifically, increases in follow‐up duration, face‐to‐face contact rates, and survey success rates were associated with decreases in the odds of a repeat incident. Furthermore, there was a marginally significant impact of complaint type on victim‐reported repeat abuse, with studies that limited eligible incidents to intimate partner violence cases reporting decreases in the odds of a repeat incident and studies including more general measures of family abuse reporting increases in these odds. It is possible that such factors have meaningful effects but that we lacked a sufficient number of studies to detect them, and we would encourage additional research on these characteristics in the future.

In interpreting our findings, we think it important to note that this body of research is characterized by a relatively small number of studies that are heterogeneous in their approaches and findings. Inevitably, our moderator analyses involved even smaller subsets of studies, creating the possibility for significant effects to be driven by a select few effect sizes. Additionally, our eligible studies suffered from a lack of geographic diversity, as only one study was conducted outside of the U.S. and two cities (New York and New Haven) accounted for six separate studies. This presents concern over the generalizability of our findings and it is possible that the impact of second responder programs varies across different environments and contexts. Thus, future research is needed to assess the findings of this review.

### 6.2 Overall completeness and applicability of evidence

Given the comprehensive nature of our search strategies, we believe that this review encompasses all experimental and quasi‐experimental evaluations examining the effect of second responder interventions on repeat family abuse published through 2019. However, our secondary search strategies, while less comprehensive, also extend this timeframe through October of 2021. The original second responders review included 10 studies, and thus the 15 studies included in this update correspond to a 50% increase in research. We also believe that the results of this update have increased applicability to criminal justice agencies and policymakers through the inclusion of additional outcomes and moderator analyses. These results, in particular, may prove important for policing agencies and social service providers attempting to address repeat family abuse, as they point to specific issues that should be considered when implementing second responder programs. We do reiterate, however, that only one included study was conducted outside of the United States, and thus the applicability of our findings to different international contexts is uncertain.

### 6.3 Quality of the evidence

Compared to many interventions in criminal justice and the social sciences, this body of research could be considered robust in methodological quality, with 40% of included studies using experimental methods. However, most studies still used quasi‐experimental methods, and very few of these studies employed statistical matching techniques (for an exception see Messing et al., 2015). The most common approach among quasi‐experimental studies involved post‐hoc comparisons of cases receiving treatment to those not receiving treatment, with the use of multi‐variate methods or some determination of face validity for comparison cases. As such, this created further variance as to whether studies employed an intent‐to‐treat approach or only analyzed cases receiving treatment. The effect of this variance in methodological quality was further indicated by the notably different results for experimental and quasi‐experimental studies, where experimental studies suggested significant increases in the odds of a police‐reported repeat family abuse incident and quasi‐experimental studies suggested notable but nonsignificant decreases in the odds of a repeat incident. Given these considerations, we feel that the quality of evidence in this review likely impacted the results of our analyses. We encourage more weight to be placed on the results of our experimental studies, as they may provide better representations of the impacts that second responder programs could be expected to produce in field settings.

### 6.4 Limitations and potential biases in the review process

We were able to calculate at least one effect size for all eligible studies and thus no studies that reported a police or victim‐reported measure of repeat family abuse were omitted from our models. We also employed a robust search strategy that included an array of both published and unpublished databases. While our study‐level risk of bias ratings were not explicitly factored into the synthesis of our results, these ratings were highly correlated with research design, which was examined across a series of moderator analyses. Additionally, there was heterogeneity in the specifics of the outcomes and interventions included in this review that could have impacted our results, however, we went to great lengths to explore this potential across a number of additional moderator analyses. Thus, there were no specific limitations or biases in our review process. Any potential sources of bias were likely small and not substantively impactful on our overall findings.

However, certain study characteristics which we employed as moderator variables, such as face‐to‐face contact rate, were not reported by several studies. This limits our ability to test and confidently conclude any potential relationships between these factors and study outcomes. Additionally, inferring the relationship between police‐reported repeat abuse and true changes in the prevalence of abuse may be tenuous. As scholars have noted, changes in this measure could be a proxy for either victim willingness to call the police or repeat abuse (Davis & Taylor, 1997; Davis et al., 2001; Hovell et al., 2006). Accordingly, accurate interpretation of this measure remains elusive, and direct measurements of victim willingness to call the police following second responder interventions may be valuable to examine in future reviews (e.g., see Koppensteiner et al., 2019; Stover et al., 2010).

### 6.5 Agreements and disagreements with other studies or reviews

The findings of this updated review are consistent with those of the prior review (Davis et al., 2008). Second responder interventions appear to increase the official reporting of repeat family abuse but not the true prevalence of these incidents. Evidence suggesting that second responder programs increase the use of domestic abuse services is also consistent with the theoretical mechanism through which these programs are proposed to address repeat abuse (Davis et al., 1997; Mazerolle et al., 2018; Messing et al., 2015). However, the lack of observed effect on victim‐reported measures of repeat abuse calls into question the connection between service use and revictimization. Finally, the findings of our moderator analysis suggesting that second responses may need to occur within a short period of time following an incident are consistent with the idea of a small window of opportunity for domestic abuse interventions to be effective (Anderson et al., 1995; Davis & Smith, 1994; see also Messing et al., 2015; Scott et al., 2015).

## 7 AUTHORS' CONCLUSIONS

### 7.1 Implications for practice and policy

Despite uncertainty as to their effects, family abuse programs that resemble the second responders model continue to be implemented and funded (see Meyer, 2011; Scott et al., 2015; Koppensteiner et al., 2019). These interventions are undoubtedly well‐intentioned, but that does not preclude them from generating unintended or even backfire effects. While repeat family abuse is a pressing criminal justice and public health issue, for which agencies may be desperate to find a solution, such programs should not be adopted based on their logic models alone, or in the absence of empirical evidence to support their efficacy. While this updated review yielded notable increases in the number of studies that were considered, it raised similar concerns about the efficacy of second responder programs to reduce repeat family abuse, and whether these programs are deserving of future funding and adoption.

Our results continue to suggest that second responder programs do not produce clear reductions in repeat abuse, but at the same time may increase the degree to which abuse is reported. While we have considered these findings as favorable to control groups, this can certainly be debated. Evidence of increased reporting to the police in the absence of increased reporting on victim surveys may indicate improvement in victims' confidence and willingness to contact police. In this regard, contact with second responders may provide external validation concerning abusive experiences that might otherwise be minimized by the victim and those around them (see Yamawaki et al., 2012), perhaps increasing the propensity for victims and family members to reach out when future incidents occur (Hovell et al., 2006). While this is not the long‐term goal of second responder programs, family abuse is a frequently underreported phenomenon and there is potential for increased police contact to lead to long‐term reductions in repeat victimization. Thus, many may consider the findings of this review to be favorable to second responder programs. Additionally, it does appear that second responder programs can maximize their chances of reducing repeat abuse by focusing on the speed of the response. That is, increases in the immediacy of the second response seem to decrease the odds of a victim‐reported repeat family abuse incident, though this does not necessarily indicate that there is any specific threshold of time that would be guaranteed to generate significant reductions in abuse. It also appears as though second responder programs may be particularly ineffective at reducing repeat abuse when measured within the same household. It is not clear why this might be the case and any implications of this finding are tenuous, though it is possible that the true potential for second responder programs to reduce family abuse is through encouraging victims to leave their abusive situations, if able, or to access services that lead to separation from their abuser. Considerably more research is needed on this relationship.

Second responder programs may produce other intended effects, however, even if these effects do not lead to observable reductions in repeat abuse. Namely, we find evidence to suggest that second responders are successful at providing family abuse victims with an increased awareness and knowledge of available services, as well as the means to access them. While this is undoubtedly a desired outcome, it is not clear why increased service uptake does not appear to lead to reductions in abuse across our studies. An important detail may be the specific types of services that victims access and the specific goals of those services. While there is research to suggest that domestic violence services can increase feelings of self‐efficacy and coping skills (Bennett et al., 2004), there is also research to suggest that certain types of services can lead to retaliatory violence. Specifically, services that reduce the long‐term exposure between victims and their abusers have been associated with decreases in violence, while those that do not have often been associated with increased abuse (see Dugan et al., 2003). While speculative, this theory would appear to fit the findings of this review. Second responder evaluations that followed the same households reported notable increases in abuse, while those that followed the same victim more generally reported notable decreases. It could be that second responder programs would benefit from an increased focus on the separation of victims and abusers, whether through the provision of services that lead to that separation or through direct encouragement of separation during the second response itself. However, it must also be recognized that abusive relationships are exceedingly complex, and that separation may not be possible or even desirable for all victims (see Hovell et al., 2006).

The composition of second responder programs, as we have defined them in this review, is also variable in nature. Several programs involved a validated lethality assessment screening, several also incorporated a dedicated focus on investigation and prosecution, while others conducted public education campaigns. While we likely did not have enough studies in each of these categories to detect any significant outcome differences, if present, these variations in design did display effects that trended in different directions. Thus, our aggregate results do not necessarily indicate that any specific type of second response program (e.g., lethality assessment, dedicated domestic violence units, etc.) is ineffective.

It is also important to note that second responder interventions are only applied to cases in which a triggering offense is reported to the police, and it is unclear how substantial any observed effects may be on re‐victimization more broadly. While the inclusion of victim surveys is intended to provide insight into this dark figure of repeat abuse, reactive criminal justice interventions are inevitably limited in their reach. This is particularly true when dealing with issues that are frequently hidden from official statistics, as victims that remain unknown to police will never be eligible to receive a second response. Accordingly, these interventions should not be viewed as a general preventative approach, and any inferences regarding their effects should be limited only to those victims who become known to law enforcement agencies.

With 15 studies now analyzed on this topic and decades of research, given the lack of observed effects for victim‐reported abuse, it seems that it may be time to move away from second responder interventions. If these programs are effective, the effect appears limited to an increased reporting of crime and increased retention of services. While both of these outcomes may be desirable, they are also of unknown long‐term efficacy, and considering the results of this updated review, policymakers may want to question whether second responder programs are worthy of investment. Alternative programs involving primary prevention with youth (e.g., programs like Safe Dates, Shifting Boundaries, and Dating Matters) have all shown effectiveness in rigorous experimental evaluations (Foshee et al., 2000; Niolon et al., 2019; Taylor et al., 2013). Overall, there may simply be better options for preventing repeat family and intimate partner abuse.

### 7.2 Implications for research

In their rapid review of criminal justice responses to domestic and family abuse, Mazerolle et al. (2018) suggested that, given the mixed evidence, multi‐agency programs such as second responders were a priority for evaluation. While our results do not support the efficacy of these programs, we provide a similar assessment. If the adoption of second responder interventions is to continue, then further research is needed across a number of domains.

First, evidence of a significant increase in police‐reported repeat abuse for experimental studies, coupled with a notable but nonsignificant decrease in these measure for quasi‐experimental studies, is suggestive of the need for more research using rigorous methodologies. While this review yielded an additional five studies, only one of these studies was experimental, and we are uncertain as to whether the divergent results we observed were solely the product of methodological quality. This need is further exacerbated by the scarcity of quasi‐experimental designs that utilized statistically matched control groups. More homogeneity in methodological quality may also allow for a stronger ability to explore moderator variables, as it may limit the degree to which certain study characteristics are correlated with research designs. On a related note, increased reporting of face‐to‐face contact rates, and/or providing both intent‐to‐treat and treatment‐only estimates, may be useful in determining the degree to which treatment crossovers impact study results (for an example see Messing et al., 2015).

As previously discussed, our moderator analyses also suggested that the immediacy of the second response may be an important determinant of treatment effects. Thus, we think it important for future studies to introduce and document variation in the timing of these responses, particularly in situations where researchers can engage in a priori assignment of cases to treatment. While one prior study did attempt to do this (Davis et al., 2010), finding little difference in the outcome between response timings, our results here suggest that this effect may not have been generalizable, and that further replications are needed. There are likely no one‐size‐fits‐all solutions to domestic abuse (see Dugan et al., 2003; Hovell et al., 2006), and what works or does not work in one context may generate different effects in another. We also encourage additional research on implementation elements such as follow‐up duration and eligible complaint types (e.g., comparison of effects between intimate partner cases and general family abuse cases), as these elements showed preliminary signs of importance but failed to reach conventional levels of statistical significance.

Our findings concerning the impact of the unit of analysis on study results also presents important implications for future work. It is possible that second responder programs reduce repeat family abuse only if they result in the victim separating from their abuser or accessing services that aim to create long‐term exposure reduction. Alternatively, it is possible that increased reporting effects are observed for multiple household members, resulting in greater contact with police across the entire familial unit. These claims are merely speculative at this point, however, and it remains unclear why these effects were observed. Future studies may benefit from incorporating more specific outcome measurements that can allow for inferences to be made regarding these mechanisms. For instance, it may be important to document whether victims separated from their abusers following a second responder intervention or accessed services that sought to create separation, even providing separate estimates of repeat abuse for these victims if possible. Future studies may also benefit from documenting who is responsible for filing the initial and follow‐up police reports. While several studies included in this review did attempt to capture some of these elements (e.g., Koppensteiner et al., 2019; Messing et al., 2015; Stover et al., 2010), increased reporting of this kind might lead to a greater understanding of the specific situations in which second responder programs do, or do not, appear to work.

Ultimately, evidence is now accumulating to suggest that second responder interventions are not an effective approach for reducing repeat family abuse. However, if these programs continue to be implemented and adopted, then there is an increasing need to research strategies capable of increasing their efficacy, or at worst, mitigating their potential backfire effects.

## ROLES AND RESPONSIBILITIES

Robert C. Davis, David Weisburd, and Bruce Taylor designed the original systematic review following Campbell Collaboration conventions and procedures. Kevin Petersen, Robert C. Davis, David Weisburd, and Bruce Taylor designed the updated review with assistance from the GPD team of Elizabeth Eggins, Lorraine Mazerolle, and Angela Higginson. The GPD team performed the primary search and sent the results to Kevin Petersen, who also performed all supplementary searches. Study screening was conducted by Kevin Petersen and Robert C. Davis, any eligibility decisions that required debate were determined by Kevin Petersen, Robert C. Davis, and David Weisburd Study coding was conducted by Kevin Petersen and another graduate research assistant. Kevin Petersen took primary responsibility for writing the report, along with help from Robert C. Davis, David Weisburd, and Bruce Taylor All effect sizes were calculated, and meta‐analytic models estimated, by Kevin Petersen, along with support for the effect sizes included in the prior review provided by Bruce Taylor

- Content: Kevin Petersen, Robert C. Davis, David Weisburd, Bruce Taylor
- Systematic review methods: Kevin Petersen, Robert C. Davis, David Weisburd, Bruce Taylor
- Statistical analysis: Kevin Petersen, Bruce Taylor
- Information retrieval: Elizabeth Eggins, Lorraine Mazerolle, Angela Higginson, Kevin Petersen, Robert C. Davis

## SOURCES OF SUPPORT

This study/project is funded by the National Institute for Health Research (NIHR) Incentive Award Scheme 2020 Reference 133290. The views expressed are those of the author(s) and not necessarily those of the NIHR or the Department of Health and Social Care.

## CONFLICT OF INTERESTS

Robert C. Davis, Bruce Taylor, and David Weisburd produced the earlier review of second responder programs. Robert C. Davis and Bruce Taylor have also conducted three of the studies previously reviewed. Kevin Petersen has not conducted evaluation research or published on the effectiveness of second responder programs. No authors have any ideological bias toward the effectiveness of second responder programs.

## Supporting information

Supporting information.

## Footnotes

^1^
It should be noted, however, that victim surveys also have limitations related to low and variable response rates (see Davis et al., 2010; Stover et al., 2010), calling into question the comparability of respondents who remain in the study and those that are lost to follow‐up. Viewing either measure in isolation can be problematic.

^2^

https://thrivenyc.cityofnewyork.us/program/crime-victim-assistance-program-in-precincts-and-psas

^3^
By similar case make‐up, we mean that the distribution of types of offenses (e.g., assault, harassment, violation of restraining order, etc.) had to be comparable between the intervention and control areas. By similar demographic make‐up, we mean that the distribution of basic demographics available on police reports (victim and abuser age, gender, and race) and from census data (indicators of concentrated disadvantage) had to be comparable between the intervention and control sites.

^4^
See https://www.cdc.gov/violenceprevention/intimatepartnerviolence/fastfact.html

^5^
These journals included *Police Quarterly, Policing and Society, Policing, Police Practice and Research, Violence against Women, Journal of Interpersonal Violence, Journal of Family Violence, Violence and Victims*, and *Criminology and Public Policy*.

^6^
Relevant reviews included Mazerolle et al. (2018), Patterson and Swan (2019), Rivas et al. (2016, 2019).

^7^
With the exception of research design, all moderator analyses used a single estimate of *τ* ^2^ for each included group. We chose to estimate *τ* ^2^ separately for experimental and quasi‐experimental studies for several reasons. One, we wished to remain consistent with the approach used in the original review (Davis et al., 2008), which estimated separate models for each. Two, we felt that there were a sufficient number of studies in both categories to warrant the estimation of separate models and that experimental studies were likely to display less heterogeneity than quasi‐experimental studies. Despite the estimation of separate models, however, we still employed the analog to the ANOVA method to test for significant differences between experimental and quasi‐experimental studies. To do so, we simply included the separate estimates of *τ* ^2^ in each study's inverse variance weight and tested for significant differences from within a fixed effect model.

^8^
Given that second responders can include specially trained police officers, social service workers, victim advocates, domestic violence counselors, etc. We initially considered examining whether effect sizes differed depending on who conducted the second response. However, after screening and coding our studies, it was determined that the majority of second responses were conducted by teams of police officers and social workers, and that there was not enough variance in these responses to examine as a potential moderator.

^9^
Messing et al. (2015) also conducted separate treated and intent‐to‐treat analyses for measures of repeat victimization. However, we were not able to derive multiple effect sizes for this outcome.

^10^
Second responder intervention do vary to the extent that the second response is announced or unannounced, however, whether a victim/household is assigned to a second response did not appear to be related to volunteer status in any of our studies. By extension, all studies reporting interview or survey measures necessitated that the victim agree or volunteer to cooperate with researchers. Information on whether second responses were announced or unannounced was not reported with enough consistency to be used as a moderator.

^11^
We note a word of caution here, however, as it was difficult at times to determine whether follow‐up measures were limited to the same family or household.

^12^
A number of elements were dropped from the Cochrane tools as they were not deemed to be particularly relevant to the research included in this review.

^13^
Note that both Mizrachi (2019) and Messing et al. (2015) evaluated a lethality assessment program (LAP). However, Messing et al. employed a comparison group of cases with similar risk assessments that occurred before the intervention, limiting the concern that there were systematic differences in the nature of treatment and control cases.

^14^
Given that two studies measured this outcome dichotomously and two measured it continuously, we also estimated a Hedge's *g* model. The results of this model were statistically significant and substantively similar (*g* = 0.05, *z* = 2.43, *p* = 0.015, *Q* = 1.18, *I* ^2^ = 0.00, *τ* ^2^ = 0.00).

^15^
This effect size was rounded to two decimal places. At three decimal places the effect size is 0.997, suggesting a 0.3% decrease in the odds of a victim‐reported repeat family abuse incident for treatment groups relative to control groups.

^16^
These were also the only main outcome models that displayed excess heterogeneity. While there was not significant between‐study heterogeneity in our combined victim‐reported repeat family abuse model, there was still excess heterogeneity as indicated by a *Q* statistic that was larger than its degrees of freedom.

^17^
Though these studies have often found the employment status of the offender to be an important determinant of the relationship between arrest and recidivism, we did not have a sufficient number of studies that reported employment information for the abuser to examine this relationship.

^18^
Studies that analyzed treatment‐only cases, or studies where all victims analyzed received at least one face‐to‐face contact, were included as 100% contact rates in this analysis.

^19^
The party responsible for filing the initial police report may represent a particularly important factor influencing study outcomes. It is possible that cases in which the original incident was reported by the victim (rather than family members, neighbors, or other third parties) have increased propensity to engage with the intervention and take action to prevent repeat abuse. Alternatively, it is possible that retaliatory violence is more likely when the victim files the initial police report, as opposed to a third party. Unfortunately, this information is difficult to ascertain and remains largely unknown. Thus, we are unable to account for these possibilities in the present analysis, though we encourage future researchers to do so.
