# Supplementary material for: Effects of second responder programs on repeat incidents of family abuse: An updated systematic review and meta‐analysis
Source: Campbell Syst Rev. 2022 Jan 28;18(1):e1217. doi: 10.1002/cl2.1217 (PMC8799917; doi:10.1002/cl2.1217)
Supplement: Supplementary file 1 — Supporting information. [file CL2-18-e1217-s001.docx]

Appendix A: narrative summaries of included studies

Casey et al. (2007):
Casey et al. (2007) describe a second responder program that partnered with the New Haven Department of Police Service and the Yale Child Study Center. Home visits were conducted by police-advocate teams within 5-7 days of reported intimate partner violence incidents in four of New Haven’s ten policing districts. The researchers used a quasi-experimental design that compared 102 arrest cases that successfully received a second response with 102 arrest cases in six control districts. The comparison cases were selected for their proximity in time to the cases receiving the intervention and their similarity across multiple matched variables including seriousness of charge, history of domestic violence, nature of victim-perpetrator relationship, number of days perpetrator was incarcerated following the target incident, age of victim and perpetrator, and ethnicity of victim and perpetrator. Casey et al. followed the same victim and defined recidivism as any domestic violence intervention by the police in the 12 months following the triggering incident. Results indicated that victims receiving the second response were significantly less likely to report a repeat incident to the police.

**Davis & Taylor (1997); Davis & Maxwell (2002); Davis et al. (2006):**

Davis and Taylor describe a second responder program jointly sponsored by New York’s

Victim Services (now Safe Horizon) with the New York Police Department. The intervention teamed a social worker with a police officer to follow up on incidents of family violence reported in targeted New York public housing projects, usually 7-14 days after an incident was reported to the police. The study also included a public education treatment which was unrelated to the second response treatment. A significant advantage of the sampling frame used in this and the other New York studies (see below) was that, because residents of public housing seldom move, researchers were able to achieve a high response rate in victim surveys. Face-to-face contact was made with victims in 85% of households assigned to the second responder intervention: in the remainder of cases, literature was left for later perusal by household members. The researchers randomly assigned 435 households to receive or not to receive a second response at the time a call for service was logged by the police (though police data was not available for all households). Repeat abuse was assessed by examining calls to the police involving the same household and using victim surveys. The surveys had a 72% response rate. Both sets of outcome data were collected six months after the triggering incident. Using an intent-to-treat approach, Davis and Taylor’s results indicated that the intervention households were more likely to report new abuse to the police, but there was no effect on victim-reported violence. Davis and Taylor also measured the use of victim services during the follow-up period, findings no significant differences between groups.

**Davis et al. (2001); Davis & Medina (2001); Davis & Maxwell (2002); Davis et al. (2006):**

Davis et al. (2001) used the same procedures as the earlier Davis and Taylor study. That

is, the study was an evaluation of the same Victim Services/New York Police Department

program; cases were randomly assigned to treatments; and a public education treatment

was included that was unrelated to the second response intervention. Like the earlier

study, Davis et al. also studied incidents generated from public housing units.

However, instead of a sampling frame defined by police reports of family abuse

incidents, Davis and Medina’s sampling frame consisted of 406 elder abuse cases (though police data were not available for all households). The authors report a face-to-face contact rate of approximately 50% with victims assigned to the second response condition. Repeat abuse was assessed at both six- and twelve-months post incident using both police reports involving the same household as well as victim surveys. The first survey had a response rate of 69% and the second survey had a response rate of 67%. Davis et al. used an intent-to-treat approach, and their results indicated significant increases in both police and victim-reported repeat abuse for the intervention group relative to the control group.

**Davis et al. (2010); Davis et al. (2007):**

Davis et al. (2010) studied a second response program run by the Redlands,

CA Police Department. For purposes of the study, households reporting eligible family

violence incidents were randomly assigned to receive either an immediate second

response, a delayed (7-day post-incident) second response, or no second response. A

specially trained female domestic violence police officer delivered the second response,

usually with another police officer. Contact with the victim was established in 84% of

the cases assigned to the second response condition. The study tracked 300 cases for six

months using police reports of new incidents between victim and perpetrator and victim

surveys. Surveys were successfully completed in 41% of the cases in the sample. The

response rate would have been far less had the researchers not used letter incentives and

home visits to elicit surveys from victims who could not be interviewed by phone. Also using an intent-to-treat approach, Davis et al.’s results suggested nonsignificant increases in both police and victim-reported repeat violence.

**Friday et al. (2006); Exum et al. (2010):**

Friday et al. (2006) evaluated a specialized domestic violence police unit implemented in Mecklenburg County, North Carolina. The unit consisted of a sergeant who screened cases for eligibility to receive the response, detectives who provided focused investigation of cases selected for response, and counselors who contacted victims to provide services. The criteria for case selection involved the lethality of the offense, and the goal of the program was both to provide support to victims as well as to increase the criminal justice response to domestic incidents. Friday et al. used a quasi-experimental design that followed the victims of 220 DV unit cases and 671 non-DV unit cases using official data sources for up to 24 months. No matching of victims or cases was conducted, but the study used multi-variate methods to control for potentially confounding factors such as the characteristics of the victim and the triggering offense. Friday et al. also estimated separate effects for all cases assigned to the DV unit and those that received a DV counselor. Both estimates indicated a decreased likelihood of any repeat incident for treatment victims, however neither effect was statistically significant.

**Greenspan et al. (2005):**

Greenspan et al. (2005) reported on a second response program in

Richmond, VA. The researchers used a quasi-experimental design with a sample of 120

family violence incidents. The intervention was an immediate response by social

workers employed by the Department of Social Services. The researchers compared

households that received a second response in two targeted precincts with family violence

cases in two precincts that did not have a second response program. The researchers note

that officers in the targeted precincts only summoned second responders in a small

proportion of cases. Since the researchers do not know the criteria that officers used in

selecting cases for second responses, it is difficult to be certain that the cases selected for

the intervention were truly comparable to cases in the control precincts. At the same

time, the researchers found that the treatment cases were similar to the general population

of cases in the city. Moreover, they compared the treatment and control conditions and

found the groups to be very similar on a series of demographic characteristics. The only

outcome that researchers report is a victim survey abuse measure gathered six months

after the trigger incident. The researchers also included only cases where victims received service. Interviews were completed with 76% of eligible victims, and results suggested marginally significant decreases in repeat abuse for treatment victims relative to control victims.

**Hovell et al. (2006):**

Hovell et al. (2006) conducted a quasi-experimental evaluation of a second response

program run by the police in cooperation with multiple community organizations in San

Diego. Responding police officers called for services of a family violence response team

after stabilizing the crime scene. The initial visit was followed up by other services

within a week of the incident. Researchers compared 307 households that received the

second response with a comparison group of 498 cases drawn from the same area the

year before the second response program started. Repeat violence was assessed by

examining records of family violence complaints that were made from sampled

households (it was not possible to match victim and perpetrator names, so address was

used as a proxy). The study did not include a survey measure of repeat violence and results indicated a significant increase in police-reported repeat violence for treatment households relative to comparison households.

**Koppensteiner et al. (2019):**

Koppensteiner et al. (2019) used an experimental design to evaluate a second responder intervention called “Project 360” implemented by the Leicestershire (UK) police force. Engagement workers operating within the police force contacted victims within 24 hours of a reported incident. The initial contact took place over the phone, but this was often followed up by face-to-face contact to provide further assistance. Koppensteiner et al. followed 510 victims assigned to treatment and 505 victims assigned to control for periods of 3-, 6-, 12-, and 24-months post-response using police-reported domestic violence data. They also surveyed 110 treatment victims and 104 control victims one month after the response (survey success rates of 21.6% and 20.6%) for measures of service usage and police satisfaction. Overall, there was a 65% engagement rate for treatment victims, though only 24.2% of victims received face-to-face meetings. Results indicated that treatment victims were more likely to report repeat abuse to the police and to access services, though these differences were nonsignificant.

**Messing et al. (2015):**

Messing et al. (2015) evaluated a lethality assessment program (LAP) implemented in seven policing districts throughout Oklahoma using a quasi-experimental design. Upon receiving a domestic violence complaint, specially trained police officers responded to the scene and administered a risk assessment screening to determine the victim’s propensity for domestic homicide. Victims who were determined to be at high risk were then offered an immediate phone call with a domestic violence advocate who conducted safety planning, provided service options, and encouraged the victims to set up a face-to-face meeting for additional services. Responding officers were also trained to provide safety planning advice to the victim on scene. Messing et al. conducted follow-up surveys with victims approximately 15 days and 7 months post-intervention and achieved survey success rates of 56% in the treatment group and 62% in the control group. This resulted in the analysis of 315 victims who received the LAP screening (202 of which agreed to speak to an advocate) and 212 victims of a similar risk level drawn from the year prior to LAP implementation. The baseline interviews provided measures of immediate service usage following the intervention, and the follow-up interviews assessed the degree of repeat victimization that the women experienced. Results suggested that treatment victims were significantly more likely to use domestic violence services, and reported significantly less frequent and severe repeat abuse, than control victims. Messing et al. also provided propensity score matched treatment-only estimates, and the results were similar to those of the intent-to-treat analyses.

**Mizrachi (2019):**

Using a quasi-experimental design, Mizrachi (2019) also analyzed a LAP program in Las Vegas (NV). The intervention appeared consistent with that of the one studied by Messing et al. (2015). Mizrachi used police incident data to track 954 domestic violence victims for a period of three years. Approximately 59% of victims received the LAP intervention and the comparison group was drawn from victims who did not receive the intervention during the same time period. No matching of victim or case characteristics was conducted, but Mizrachi used multivariate models to control for various characteristics of the victim, the incident, and other risk factors. Repeat violence consisted of any subsequent incidents reported to police where the original victim was once again listed as a domestic violence victim. Results indicated that the LAP intervention was associated with a significant decrease in the odds of a repeat incident.

**Pate et al. (1992):**

Pate et al (2002) conducted an experimental evaluation of a second response

program in Miami as part of the SARP replication of the Minneapolis domestic violence

arrest experiment. The second response treatment was independent of the arrest

treatment, and involved a home visit made by a domestic violence detective within 72

hours of the family violence complaint. In ninety-five percent of households assigned to

the second response condition, face-to-face contact was established with the victim, a

figure significantly higher than in the New York experiments. The study tracked 907

family violence complaints for six months and gathered information on new complaints

made to the police involving the same victim and perpetrator as well as surveys of victims.

Victim surveys had a 65% response rate. Using an intent-to-treat approach, results indicated nonsignificant differences in both police and victim-reported repeat violence.

**Regoeczi & Hubbard (2018):**

Regoeczi and Hubbard (2018) used a quasi-experimental design to assess a specialized domestic violence unit in Cleveland, Ohio. The unit involved a team of police officers and victim advocates that responded to domestic violence incidents in attempt to further investigate cases and provide victim services. The intervention also involved the use of specialized domestic violence prosecutors and a dedicated domestic violence court docket. Regoeczi and Hubbard assessed repeat domestic violence by following all cases assigned to the DV unit from July to December of 2008 to all cases not assigned to the DV unit. The unit was active in only three of five police districts in Cleveland, and thus control cases were taken from the two districts where the intervention was not implemented. Regoeczi and Hubbard followed 1,279 offenders for a period of two to two and a half years using official police data. Results indicated that there was no significant difference in the likelihood of committing a repeat domestic offense between groups.

**Stover et al. (2009):**

Stover et al. (2009) studied the same New Haven intervention described above in Casey et al. using a similar quasi-experimental design that compared domestic violence victims in five New Haven police districts that housed second response teams with five that did not. As in the Casey et al study, the sampling frame consisted of intimate partner cases in which an arrest had been made. One difference in the intervention was that Stover et al report that the second response was delivered within 72 hours of the incident while Casey et al report that the intervention was delivered 5-7 days afterwards. The researchers analyzed 512 victims for any repeat domestic violence over a 12-month follow-up period. No matching was reported to equate second response and control groups on initial characteristics, but the analyses introduced covariates including victim ethnicity, nature of charge, substance abuse, and arrest history. The authors also separated their sample by engagement levels, including victims where contact was unsuccessful, those who received less than 20 minutes of contact with second responders (low dosage), and those that received at least 20 minutes of contact (high dosage). Results indicated that high dosage victims were significantly more likely to report a repeat incident to police, but that there were no significant differences for no contact or low dosage groups.

**Stover et al. (2010):**

Stover et al. (2010) conducted another evaluation of the New Haven second

responder program reported in Casey et al. and the earlier Stover et al study. This study represented the survey component of the 2009 study, and the sampling frame included 107 domestic violence victims surveyed at 1, 6, and 12 months after the triggering incident. The recidivism measure consisted of any new incidents reported on these victim surveys, and also included a measure of service use during the one-month follow-up period. No matching was reported to equate second response and control groups on initial characteristics, but the analyses introduced covariates including victim ethnicity and perpetrator criminal history. Results suggested that there were no significant differences between groups on the frequency and severity of repeat abuse, though treatment victims did access significantly more services.

**Taylor (n.d.); Davis & Maxwell (2002); Davis et al. (2006):**

In an unpublished study, Taylor conducted another evaluation of the Victim

Services/NYPD model again using a true experimental design. This study used a sample

of 197 arrest cases drawn from family violence incidents in public housing units (though police data was not available for all households). As in the other New York studies, the second response was delivered by a social worker/police officer team 7-14 days after the initial patrol response. Police reports involving the same household were collected and victim surveys conducted six months after the trigger incident. This study had only a 44% survey success rate. Results indicated no significant differences between treatment and control groups for either police or victim-reported repeat abuse.

Appendix B: GPD Systematic Search Strategy^[[1]](#footnote-1)^

### Search Terms

To ensure optimum sensitivity and specificity, the GPD search strategy utilises a combination of free-text and controlled vocabulary search terms. Because controlled vocabularies and search capabilities vary across databases, the exact combination of search terms and field codes are adapted to each database. Final search syntax for each location will be reported in the final review.

The free-text search terms for the GPD are provided in Table 1 and are grouped by substantive (i.e., some form of policing) and evaluation terminology. Although the search strategy may vary slightly across search locations, it follows a number of general rules:

- Search terms are combined into search strings using Boolean operators “AND” and “OR”. Specifically, terms within each category are combined with “OR”, and categories will be combined with “AND”. For example: (police OR policing OR “law#enforcement”) AND (analy* OR ANCOVA OR ANOVA OR …).
- Compound terms (e.g., law enforcement) are considered single terms in search strings by using quotation marks (i.e., “law*enforcement”) to ensure that the database searches for the entire term rather than separate words.
- Wild cards and truncation codes are used for search terms with multiple iterations from a stem word (e.g., evaluation, evaluate) or spelling variations (e.g., evaluat* or randomi#e).
- If a database has a controlled vocabulary term that is equivalent to “POLICE”, the term is combined in a search string that includes both the policing and evaluation free-text search terms. This approach ensures that the search retrieves documents that do not use policing terms in the title/abstract but have been indexed as being related to policing in the database. An example of this approach is the following search string: (((SU: “POLICE”) OR (TI,AB,KW: police OR policing OR “law*enforcement”)) AND (TI,AB,KW: intervention* OR evaluat* OR compar* OR …)).
- For search locations with limited search functionality, a broad search that uses only the policing free-text terms is implemented.
- Multidisciplinary database searches are limited to relevant disciplines (e.g., include social sciences but exclude physical sciences).
- Search results are refined to exclude specific types of documents that are not suitable for systematic reviews (e.g., newspapers, front/back matter, book reviews).

#### Table A1. Free-text search terms for the GPD systematic search

| **Policing Search Terms** | **Evaluation Search Terms** | | | |
| --- | --- | --- | --- | --- |
| police  policing  “law*enforcement”  constab*  detective*  sheriff* | analy*  ANCOVA  ANOVA  “ABAB design”  “AB design”  baseline  causa*  “chi#square”  coefficient*  “comparison condition*”  “comparison group*”  “control condition*”  “control group*”  correlat*  covariat*  “cross#section*” | data  effect*  efficacy  eval*  experiment*  hypothes*  impact*  intervent*  interview*  longitudinal  MANCOVA  MANOVA  “matched group”  measure*  “meta-analy*”  “odds#ratio* | outcome*  paramet*  “post-test”  posttest  “post test”  predict*  “pre-test”  pretest  program*  “propensity score*”  quantitative  “quasi#experiment*”  questionnaire*  random*  RCT  regress* | result*  “risk#ratio*”  sampl*  “standard deviation*”  statistic*  studies  study  survey*  “systematic review*”  “t#test*”  “time#series”  treatment*  variable*  variance |

### Search Locations

To reduce publication and discipline bias, the GPD search strategy adopts an international scope and involves searching for literature across a number of disciplines (e.g., criminology, law, political science, public health, sociology, social science and social work). The search captures a comprehensive range of published (i.e., journal articles, book chapters, books) and unpublished literature (e.g., working papers, governmental reports, technical reports, conference proceedings, dissertations) by implementing a search strategy across bibliographic/academic, grey literature, and dissertation databases or repositories.

It is noted that there is substantial overlap of the content coverage between many of the databases. Therefore, the *Optimal Searching of Indexing Databases* (OSID) computer program (Neville & Higginson, 2014) has been used to analyse the content crossover for all databases that have accessible content coverage lists. OSID analyses the content coverage and creates a search location solution that provides the most comprehensive coverage via the least number of databases. Another advantage of using OSID when designing a search strategy is the reduction in the number of duplicates that would need to be removed prior to the screening phase. Databases with >10 unique titles are searched in full, whereas databases with ≤10 unique titles were searched only the unique titles and any non-serial content (e.g., reports, conference proceedings). Where a modified search of a database would be more labour-intensive than a full search and export results, a full search of the database is conducted. The final search locations and solutions are reported in Table 2.

#### Table A2. GPD search locations and protocol (January 1^st^ 1950 – December 2019)

| **INDEXED & ACADEMIC DATABASES** |  | **CONTENT COVERAGE FED INTO OSID?** | **FULL OR MODIFIED SEARCH?** | **SEARCH MODIFICATIONS** |
| --- | --- | --- | --- | --- |
| **ProQuest** | Criminal Justice | Yes | Full | None. |
|  | Dissertation and Theses Database Global | Not Available | Modified | Social Sciences subset. |
|  | Political Science | Yes | Full | None. |
|  | Periodical Archive Online | Yes | Full | None. |
|  | Research Library | Yes | Modified | Social Sciences subset. |
|  | Social Science Journals | Yes | Full | None. |
|  | Sociology | Yes | Modified | Search 2 unique journal titles and non-serial content only. |
|  | Applied Social Sciences Index and Abstracts | Yes | Full | None. |
|  | International Bibliography of the Social Sciences | Yes | Full | None. |
|  | Public Affairs Information Service | Yes | Full | None. |
|  | Social Services Abstracts | Yes | Modified | Search 5 unique journal titles and non-serial content only. |
|  | Sociological Abstracts | Yes | Full | None. |
|  | Worldwide Political Sciences Abstracts | Yes | Modified | Search 9 unique journal titles and non-serial content only. |
| **EBSCO** | Academic Search Premier | Yes | Full | None. |
|  | Criminal Justice Abstracts | Yes | Full | None. |
|  | EconLit | Yes | Full | None. |
|  | MEDLINE with Full-Text | Yes | Full | None. |
|  | Social Sciences Full-Text | Yes | Full | None. |
| **OVID** | International Political Science Abstracts | Not Available | Full | None. |
|  | PsycARTICLES | Yes | Modified | Search 4 unique journal titles only. |
|  | PsycEXTRA | Not Available | Full | None. |
|  | PsycINFO | Yes | Full | None. |
|  | Social Work Abstracts | Not Available | Full | None. |
| **Web of Science** | Current Contents Connect – Social and Behavioural Sciences Edition | Yes | Modified | Search 1 unique journal title and non-serial content only. |
|  | Book Citation Index (Social Sciences and Humanities) | Not Available | Full | None. |
|  | Conference Proceedings Citation Index (Social Sciences and Humanities) | Not Available | Full | None. |
|  | Social Science Citation Index | Yes | Full | None. |
| **Informit** | Australian Attorney General Information Service | Yes | Full | None. |
|  | Australian Criminology Database (CINCH) | Yes | Full | None. |
|  | Australian Federal Police Database | Yes | Full | None. |
|  | Australian Public Affairs Full-Text | Yes | Full | None. |
|  | DRUG | Yes | Full | None. |
|  | Health & Society Database | Yes | Modified | Search unique journal titles and non-serial content only. |
|  | Humanities and Social Sciences Collection | Yes | Full | None. |
| **Gale-Cengage** | Expanded Academic ASAP | Yes | Full | None. |
| **STANDALONE & OPEN ACCESS DATABASES** | Cambridge Journals Online | Yes | Modified | Search 4 unique journal titles in Law and Political Science collections and full search of Social Studies collection. |
|  | Directory of Open Access Journals | Yes | Full | None. |
|  | HeinOnline | Yes | Modified | Law Journals Online collection only. |
|  | JSTOR | Yes | Modified | Search unique titles across the Law, Political Science, Public Health, Public Policy, Social Work and Sociology collections only. The Criminal Justice collection had no unique content and so will be excluded from the search. Only 10% of content in this database have abstracts and a full-text search returns >250,000 results because of inability to construct complex search strings. Therefore, a modified search of the unique titles across these collections will be more pragmatic than a full search of the database. |
|  | Oxford Scholarship Online | Yes | Full | None. |
|  | Sage Journals Online and Archive (Sage Premier) | Yes | Modified | Search 5 unique journal titles and non-serial content only. |
|  | ScienceDirect | Yes | Full | None. |
|  | SCOPUS | Yes | Full | None. |
|  | SpringerLink | Yes | Full | Although this database has low uniqueness when combined with the full set of databases, a full search using only the policing search terms will be more pragmatic than a modified search on unique titles because of the restricted search functionality of this database. |
|  | Taylor & Francis Online | Yes | Modified | Although this database has low uniqueness when combined with the full set of databases, a full search using only the policing search terms will be more pragmatic than a modified search on unique titles because of the restricted search functionality of this database. |
|  | Wiley Online Library | Yes | Full | None. |
|  | California Commission on Peace Officer Standards & Training Library | No | Full | None. |
|  | Cochrane Library | No | Full | None. |
|  | CrimeSolutions.gov | No | Full | None. |
|  | Database of Abstracts of Reviews of Effectiveness (DARE) | No | Full | None. |
|  | FBI – The Fault (Reports and Publications) | No | Full | None. |
|  | Evidence-Based Policing Matrix | No | Full | None. |
|  | International Initiative for Impact Evaluation Database (3ie) | No | Full | None. |
|  | National Criminal Justice Reference Service | No | Full | None. |
|  | Safety Lit Database | No | Full | None. |
|  | Australian Institute of Criminology | No | Full | None. |
|  | Bureau of Police Research and Development (India) | No | Full | None. |
|  | Canadian Police Research Catalogue | No | Full | None. |
|  | Centre for Problem-Oriented Policing | No | Full | None. |
|  | College of Policing (including POLKA and Crime Reduction Toolkit) | No | Full | None. |
|  | European Police College (CEPOL) | No | Full | None. |
|  | Evidence for Policy and Practice Information and Coordinating Centre | No | Full | None. |
|  | National Research Institute of Police Science (Japanese) | No | Full | None. |
|  | Office of Community Oriented Policing Services | No | Full | None. |
|  | Police Executive Research Forum (US) | No | Full | None. |
|  | Police Foundation (US) | No | Full | None. |
|  | Tasmania Institute of Law Enforcement Studies (Australia) | No | Full | None. |
|  | Policing Online Information System (POLIS, Europe) | No | Full | None. |
|  | Scottish Institute for Policing Research | No | Full | None. |
|  | Centre of Excellence in Policing and Security (Australian, now archived) | No | Full | None. |

Appendix C: GPD Systematic Compilation Strategy

### Inclusion Criteria

Each record captured by the GPD systematic search must satisfy all inclusion criteria to be included in the GPD: timeframe, intervention and research design. There are no restrictions applied to the types of outcomes, participants, settings or languages considered eligible for inclusion in the GPD.

#### Types of interventions

Each document must contain an impact evaluation of a policing intervention. Policing interventions are defined as some kind of a strategy, program, technique, approach, activity, campaign, training, directive, or funding/organisational change that involves police in some way (other agencies or organisations can be involved). Police involvement is broadly defined as:

- Police initiation, development or leadership
- Police are recipients of the intervention or the intervention is related, focused or targeted to police practices
- Delivery or implementation of the intervention by police

#### Types of study designs

The GPD includes quantitative impact evaluations of policing interventions that utilise randomised experimental (e.g., RCTs) or quasi-experimental evaluation designs with a valid comparison group that does not receive the intervention. The GPD includes designs where the comparison group receives ‘business-as-usual’ policing, no intervention or an alternative intervention (treatment-treatment designs).

The specific list of research designs included in the GPD are as follows:

- Systematic reviews with or without meta-analyses
- Cross-over designs
- Cost-benefit analyses
- Regression discontinuity designs
- Designs using multivariate controls (e.g., multiple regression)
- Matched control group designs with or without pre-intervention baseline measures (propensity or statistically matched)
- Unmatched control group designs with pre-post intervention measures which allow for difference-in-difference analysis
- Unmatched control group designs without pre-intervention measures where the control group has face validity
- Short interrupted time-series designs with control group (less than 25 pre- and 25 post-intervention observations)
- Long interrupted time-series designs with or without a control group (≥25 pre- and post-intervention observations)
- Raw unadjusted correlational designs where the variation in the level of the intervention is compared to the variation in the level of the outcome

The GPD excludes single group designs with pre- and post-intervention measures as these designs are highly subject to bias and threats to internal validity.

### Systematic Screening

To establish eligibility, records captured by the GPD search progress through a series of systematic stages which are summarised in Figure C1, with additional detail provided in the following subsections. All research staff working on the GPD undergo standardised training before beginning work within any of the stages detailed below. Staff then complete short training simulations to enable an assessment of their understanding of the GPD protocols and highlight any areas for additional training. In addition, random samples of each staff’s work are regularly cross-checked to ensure adherence to protocols. Disagreements about screening decisions between staff are mediated by either the project manager or GPD chief investigators.

#### Title and abstract screening

After removing duplicates, the title and abstract of records captured by the GPD systematic search is screened by trained research staff to identify potentially eligible research that satisfies the following criteria:

- Document is dated between 1950 – present
- Document is unique (i.e., not a duplicate)
- Document is about police or policing
- Document is an eligible document type (e.g., not a book review)

Records are excluded if the answer to any one of the criteria is unambiguously ‘No’, and will be classified as potentially eligible otherwise. Records classified as eligible at the title and abstract screening stage progress to full-text document retrieval and screening stages.

#### Full-text eligibility screening

Wherever possible, a full-text electronic version of an eligible record is imported into *SysReview* (review management software; Neville & Higginson, 2014). For records without an electronic version, a hardcopy of the record is located to enable full-text eligibility screening. The full-text of each document is screened to identify studies that satisfy the following criteria:

- Document is dated between 1950 – present
- Document is unique
- Document reports a quantitative statistical comparison
- Document reports on policing evaluation
- Document reports in a quantitative impact evaluation of a policing intervention
- Evaluation uses an eligible research design

***Figure C1.*** GPD systematic compilation process

Appendix D: Second responders meta-analysis coding sheet

**Reference Information**

1. Document ID: __ __ __ __

2. Study author(s): ____________________

3. Study title: _______________________

4a. Publication type: ______

1. Book

2. Book chapter

3. Journal article (peer reviewed)

4. Thesis or doctoral dissertation

5. Government report (state/local)

6. Government report (federal)

7. Police department report

8. Technical report

9. Conference paper

10. Other (specify)

4b. Specify (Other)_____________________

5. Publication date (year): ______________

6a. Journal Name: ____________________

6b. Journal Volume: _______________

6c. Journal Issue: ____________

7. Date range of research (when research was conducted):

Start: ____________

Finish: ____________

8. Source of funding for study: ___________________

9. Country of publication: ___________________

10. Date coded: ___________

11. Coder’s Initials: __ __ __

**Describing the Sample**

12. What types of incidents were eligible? (Select all that apply)

1. Intimate partner cases

2. Family abuse cases

3. Elder abuse cases

4. Other (specify)

12b. Specify (Other) _____________

13. What kinds of criminal charges were eligible? (Select all that apply)

1. Assault

2. Harassment

3. Menacing

4. Violation of restraining order

5. Other (specify)

13b. Specify (Other) _____________

14. What type police responses were eligible?

1. Cases in which an arrest was made

2. Cases in which crime complaints were filed

3. Any report, founded or unfounded

4. Other (specify)

14b. Specify (Other) ___________

14c. What recruitment method was used to select cases?

1. Assignment of official reports/calls for service

2. Assignment by volunteer status

3. Other

14d. Specify (Other) ______________

15**.** Other than the factors described in 12-14, describe any restrictions on selecting cases for the sample: Is there reason to think that the sample is not representative of all DV complaints within the jurisdiction studied? _______________________________________

16. Characteristics of victims in the sample (enter N/I for not included)

a. Average age _______

b. Gender

1. Percent female ________ %

2. Percent male _________ %

c. Education

1. High school grad _______ %

2. Did not graduate high school ______ %

d. Currently employed

1. Employed full time _____ %

2. Employed part-time _____ %

3. Not employed ______ %

e. Residence type

1. Own home ______ %

2. Rent _______ %

3. Public housing _______ %

17. Characteristics of perpetrators in the sample (enter N/I for not included)

a. Average age _______

b. Gender

1. Percent female ________ %

2. Percent male _________ %

c. Education

1. High school grad _______ %

2. Did not graduate high school ______ %

d. Currently employed

1. Employed full time _____ %

2. Employed part-time _____ %

3. Not employed ______ %

e. Residence type

1. Own home ______ %

2. Rent _______ %

3. Public housing _______ %

**Describing the Response**

18. What did home visits consist of? (Select all that apply)

1. Assess victim’s current situation and history of abuse in relationship

2. Develop safety plan with victim

3. Discuss nature of abuse

4. Assess victim needs

5. Provide information and referrals to service programs

6. Interact with abuser

7. Other

18b. Specify (Other)___________________

19. Who was involved in the implementation of the response? (Select all that apply)

1. Domestic/family violence police officer

2. Police victim caseworker

3. Prosecutor victim caseworker

4. Independent victim advocate

5. Other (specify)

19b. Specify (Other)___________________

20. How long after the incident was reported was the second response attempted?

1. Immediate or within 72 hours

2. Within several days of incident (7 or less)

3. More than several days after incident (more than 7)

4. Other (specify)

20b. Specify (Other)___________________

20c. If provided, what was the average number of hours that elapsed between the incident and the second response attempt? ________________

21**.** Is information provided on the average length of visits?

1. No

2. Yes 🡺 Average length: ______________________________

22. Was the visit unannounced, or was there an attempt made to call the victim first?

1. Visits were typically unannounced

2. Phone contact attempted prior to visit

3. Other (specify)

23. Is information available on how often perpetrators were present during visit?

1. No

2. Yes 🡺 % of cases: ________________________________

23b. Specify (Other)___________________

24. Did the second response program exist prior to the evaluation, or was program implemented in conjunction with the evaluation?

1. Program implemented in conjunction with evaluation

2. Program existed prior to evaluation 🡺 For how long? __________________

25. Does study indicate that author(s) had a relationship with the program prior to the evaluation?

1. No indication of prior relationship

2. Paper indicates prior relationship (describe: ___________________________)

***Implementation of Response***

26. In what proportion of targeted households did the second responders establish face-to-face contact with the victim? _____ %

27. If face-to-face contact could not be established, what did the intervention consist of (e.g., literature or letter left; phone call):

________________________________________________________________________________________________________________________________________________________________________________________________________________________

***Location of the intervention***

28. Country where study was conducted: __________________

29. City (and state/province, if applicable) where study was conducted: _________________

*The following questions refer to the area receiving treatment:*

30. Geographic area receiving treatment: ______

1. Micro place (street segments/blocks)/ public housing development

2. Neighborhood/police beat

3. Police district/precinct

4. Entire city

5. Other (specify)

30b. Specify (Other)___________________

31. What is the exact geographic area receiving treatment? ________________________________________________________________________

*The following refer to the area not receiving treatment (applicable if there is a separate control group in the study)*

32. Was comparison group drawn from different geographic area than treatment group?

1. No ==> *Skip to Q 35*

2. Yes

33a. Geographic area NOT receiving treatment: ______

1. Micro place (street segments/blocks)/public housing development

2. Neighborhood/police beat

3. Police district/precinct

4. Entire city

5. Other (specify)

6. N/A (no control area)

33b. Specify (Other)___________________

34. What is the exact geographic area not receiving treatment? ________________________________________________________________________

***Confounding interventions***

35a. Was the second response treatment confounded with any other interventions (e.g., enhanced evidence collection or prosecution)?

1. Yes

2. No

35b. If yes, describe: _______________________________________________

**Methodology/Research design:**

36. Type of study:

1. Randomized experiment ==>

36a. How were cases randomized?___________________________

____________________________________________________

36b. What was the rate and cause of experimental misassignments? _______________ ___________________________________

___________________________________________________

36c. Were misassignments analyzed as assigned or as treated?

1. Analyzed as assigned
2. Analyzed as treated
3. Nonequivalent control group (quasi-experimental) ==> 36d. How were

control cases selected?

1. Matched cases (method of matching:_________________________)
2. According to objective case criteria (specify:__________________)
3. Based on possibly unbiased variable (e.g., time of day, precinct)
4. Selected by staff conducting intervention using subjective criteria
5. Treatment refusers or drop-outs
6. Other (Specify: _________________________________________)

37. If more than one treatment or comparison group used, describe nature of each:

Treatment groups Comparison groups

38a. Were efforts made to determine similarity in case, victim, and/or perpetrator criteria between treatment and comparison groups?

1. No

2. Yes 🡺 31b. What were the results? _________________________________

____________________________________________________________

____________________________________________________________

39. Did researchers believe any baseline differences biased study results? If so, in what direction? _______________________________________________________________

_______________________________________________________________

39b. What was the unit of analysis?

1. Same pairing/household

2. Same victim

3. Same offender

***Outcomes reported***

40. Which outcome measures are reported in the study? (Select all that apply)

Same victim as Any

original incident victim

1. New family violence incident reports to the police ____ _____

2. New family violence arrests ____ _____

3. New arrests for any offense ____ _____

4. New abuse (from victim survey)

5. Satisfaction with police response (from victim survey)

6. Willingness to report future incidents (from victim survey)

7. Awareness/use of victim services (from victim survey)

40b. Specify (other) _________

40c. What was the follow-up period for the intervention? ________

1. 6 months or less

2. 1 year or less

3. More than 1 year

4. Multiple follow-up periods or other (specify)

40d. Specify (Other) ______

**NOTE: COMPLETE ITEMS 41-60 FOR EACH OUTCOME MEASURE REPORTED (AND FOR EACH SET OF CONTROL-TREATMENT COMPARISONS, IF MORE THAN ONE CONTROL OR TREATMENT GROUP)**

41a. Document ID: ______________

41b. Outcome number for current study: ___________________

42a. What is the specific outcome being captured on this coding form? __________

42b. If victim surveys were used, what was response rate? ________ %

43. Did the researcher assess the quality of the data collected?

1. Yes

2. No

44. Did the researcher(s) express any concerns over the quality of the data?

1. Yes

2. No

44b. If yes, explain ____________________________________________________________________________________________________________________________________________________________

**Effect size/Reports of statistical significance**

*Dependent Measure Descriptors*

***Sample size***

45. What is the total sample size in the analysis? ________

46. What is the total sample size of the treatment group (group that receives the response)? _______

47. What is the total sample size of the control group? _____

48. Did the analysis include all cases assigned to treatment or only those with whom face-to-face contact was established by the home visit team?

1. All cases assigned to treatment

2. Only cases where victim was home at time of visit

***Effect Size Data***

49. Raw difference favors (i.e. shows more success for):

1. Treatment group

2. Control group

3. Neither (exactly equal)

9. Cannot tell (or statistically insignificant report only)

50. Did a test of statistical significance indicate statistically significant differences between the control and treatment groups?

1. Yes

2. No

3. Can’t tell

4. N/A (no testing completed)

51. Was a standardized effect size reported?

1. Yes

2. No

52. If yes, what was the effect size? ______

53. If yes, page number where effect size data is found ________

54. If no, is there data available to calculate an effect size?

1. Yes

2. No

55. Type of data effect size can be calculated from:

1. Means and standard deviations

2. *t*-value or *F*-value

3. Chi-square (df=1)

4. Frequencies or proportions (dichotomous)

5. Frequencies or proportions (polychotomous)

6. Other (specify)

55b. Specify (other) _________

*Means and Standard Deviations*

56a. Treatment group mean. _____

56b. Control group mean. _____

57a. Treatment group standard deviation. _____

57b. Control group standard deviation. _____

*Proportions or frequencies*

58a. *n* of treatment group with a successful outcome. _____

58b. *n* of control group with a successful outcome. _____

59a. Proportion of treatment group with a successful outcome. _____

59b. Proportion of control group with a successful outcome. _____

*Significance Tests*

60a. *t*-value _____

60b. *F*-value _____

60c. Chi-square value (*df*=1) _____

*Calculated Effect Size*

61. Effect size ______

**Conclusions made by the author(s)**

62. Conclusion about the impact of the intervention?

1. The authors conclude abuse declined

2. The authors conclude abuse did not decline

3. Unclear/no conclusion stated by authors

63. Did the author(s) conclude that the second responder intervention was beneficial?

1. Yes

2. No

3. Can’t tell

64. Did the author(s) conclude there a relationship between the treatment and a reduction in abuse? _____

1. Yes

2. No

3. Can’t tell

65. Additional notes about conclusions:

____________________________________________________________________________________________________________________________________________________________

Appendix E: Effect size formulas and methods

Several methods were used to generate effect sizes for our eligible studies. This appendix documents and details each of these methods, the studies that each method was used for, and the formula/function used (where applicable). The methods presented are ordered based on the sequence in which the functions where written (i.e., some functions nest earlier functions within them). The effect sizes and functions displayed were created in R statistical software (R Core Team, 2020). All formulas follow those presented in Lipsey and Wilson (2001), and the results of all calculations were verified against equivalent calculations using David Wilson’s effect size calculator (<https://www.campbellcollaboration.org/escalc/html/EffectSizeCalculator-Home.php>).

**1. 2x2 Cell Frequencies:**

When given 2x2 cell frequencies, we calculated logged odds ratios and the variance of the logged odds ratios using the standard cross-product of the cells, as follows:

$$\ln\left( OR \right)=ln\left( \frac{ad}{bc} \right)$$

$$Vln\left( OR \right)= \frac{1}{a}+\frac{1}{b}+\frac{1}{c}+\frac{1}{d}$$

The R functions written for these formulas were:^[[2]](#footnote-2)^

logOR_counts <- **function**(a, b, c, d) {

log((a*d)/(b*c))

}

logVOR_counts <- **function**(a, b, c, d) {

(1/a + 1/b + 1/c + 1/d)

}

Effect sizes using these formulas were calculated for the following study/outcome:

- Casey et al. (2007) – Repeat abuse using official data

**2. Proportion of successes/failures:**

When given proportions of successes/failures in each group, we calculated logged odds ratios and the variance of the logged odds ratio by using these proportions to calculate the cell frequencies, as follows:

$$a=p1*n1$$

$$b=n1-a$$

$$c=p2*n2$$

$$d=n2-c$$

The R functions written for these formulas calculate the cell frequencies and then call the formula written in method 1:

logOR_prop <- function(p1, n1, p2, n2) {

a <- (p1*n1)

b <- (n1 - a)

c <- (p2*n2)

d <- (n2 - c)

logOR_counts(a, b, c, d)

}

logVOR_prop <- function(p1, n1, p2, n2) {

a <- (p1*n1)

b <- (n1 - a)

c <- (p2*n2)

d <- (n2 - c)

logVOR_counts(a, b, c, d)

}

Effect sizes using these formulas were calculated for the following studies/outcomes:

- Davis et al. (2001) – Repeat abuse using official data
- Davis et al. (2001) – Repeat abuse using unofficial data
- Davis & Taylor (1997) – Repeat abuse using official data
- Davis & Taylor (1997) – Repeat abuse using unofficial data
- Taylor (unpublished) – Repeat abuse using official data
- Taylor (unpublished) – Repeat abuse using unofficial data
- Davis et al. (2010) – Repeat abuse using official data
- Davis et al. (2010) – Repeat abuse using unofficial data
- Greenspan et al. (2005) – Repeat abuse using unofficial data
- Koppensteiner et al. (2019) – Repeat abuse using official data
- Koppensteiner et al. (2019) – Victim use of services

**3. Hedge’s *g* conversion to odds ratio using Cox logit method:**

A small number of studies required us to calculate standardized effect sizes. The formula for these computations were as follows:

$$d=\frac{\bar{x}_{1}- \bar{x}_{2}}{\sqrt{\frac{{s_{1}}^{2}\left( n_{1}-1 \right)+ {s_{2}}^{1}(n_{2}-1)}{n_{1}+ n_{2}-2}}}$$

$$j=1-\frac{3}{4N}$$

$$g=d*j$$

$$v_{g}= \frac{n_{1}+ n_{2}}{n_{1}*n_{1}}+\frac{g^{2}}{2*(n_{1}+n_{2})}$$

With standardized mean differences calculated, we then approximated logged odds ratios using the Cox logit method:

$$OR=d*1.65$$

$$v_{OR}= \frac{v_{g}}{0.367}$$

The R functions written for these formulas first calculate *d, j*, and then finally *g*. The function for the variance of the standardized mean difference requires the formula used to calculate the standardized mean difference to be written first:

Hedges_g <- function(mean1, mean2, sd1, sd2, n1, n2, N) {

d <- (mean1 - mean2)/sqrt((sd1^2*(n1 - 1) + sd2^2*(n2 - 1))/(n1 + n2 - 2))

j <- 1 - (3/(4*N - 9))

d*j

}

VHedges_g <- function(mean1, mean2, sd1, sd2, n1, n2, N) {

((n1 + n2)/(n1*n2)) + (Hedges_g(mean1, mean2, sd1, sd2, n1, n2, N)^2/(2*(n1 + n2)))

}

logOR_Cox <- function(g) g*1.65

logVOR_Cox <- function(vg) vg/.367

Effect sizes using these formulas were calculated for the following studies/outcomes:

- Davis & Taylor (1997) – Victim use of services
- Stover et al. (2010) – Repeat abuse using unofficial data
- Stover et al. (2010) – Victim use of services
- Messing et al. (2015) – Repeat abuse using unofficial data

**4. Taking adjusted odds ratios directly from regression models**

Effects sizes could be directly taken from regression models for the following studies/outcomes:

- Hovell et al. (2006) – Repeat abuse using official data
- Pate et al. (1992) – Repeat abuse using official data
- Pate et al. (1992) – Repeat abuse using unofficial data
- Stover et al. (2009) – Repeat abuse using official data
- Friday et al. (2006) – Repeat abuse using official data
- Messing et al. (2015) – Victim use of services
- Mizrachi (2019) – Repeat abuse using official data

**5. Deriving odds ratio from chi-square**

One study (Regoeczi & Hubbard, 2018) required us to calculate a standardized mean difference using the results of a chi-square test. We then converted this standardized mean difference to a logged odds ratio.

First, we used David Wilson’s effect size calculator to calculate a standardized mean difference (Cohen’s *d*) using the reported sample size and the chi-square value. We then manually applied the Hedge’s *g* correction to the standardized mean difference using the *j* and *g* equations from method 3 above. Next, we calculated the variance of our Hedge’s *g* value manually as follows:

$$v_{g}= \frac{g^{2}}{\chi^{2}}$$

Finally, we used the Cox logit conversion function reported in method 3 to calculate the logged odds ratio and its associated variance.

1. Appendices B and C are taken directly from Higginson, A., Eggins, E., Mazerolle, L. and Stanko, E. (2015). *The Global Policing Database [Database and Protocol].* [↑](#footnote-ref-1)
2. Built-in functions such as “escalc” in the *metafor* package also have much of the functionality of the manually constructed functions shown here (see Viechtbauer, 2010). [↑](#footnote-ref-2)
